# Patients with Alzheimer’s disease dementia show partially preserved parietal ‘hubs’ modeled from resting-state alpha electroencephalographic rhythms

**DOI:** 10.3389/fnagi.2023.780014

**Published:** 2023-01-26

**Authors:** Susanna Lopez, Claudio Del Percio, Roberta Lizio, Giuseppe Noce, Alessandro Padovani, Flavio Nobili, Dario Arnaldi, Francesco Famà, Davide V. Moretti, Annachiara Cagnin, Giacomo Koch, Alberto Benussi, Marco Onofrj, Barbara Borroni, Andrea Soricelli, Raffaele Ferri, Carla Buttinelli, Franco Giubilei, Bahar Güntekin, Görsev Yener, Fabrizio Stocchi, Laura Vacca, Laura Bonanni, Claudio Babiloni

**Affiliations:** ^1^Department of Physiology and Pharmacology “Vittorio Erspamer”, Sapienza University of Rome, Rome, Italy; ^2^IRCCS Synlab SDN, Naples, Italy; ^3^Neurology Unit, Department of Clinical and Experimental Sciences, University of Brescia, Brescia, Italy; ^4^Clinica Neurologica, IRCCS Ospedale Policlinico San Martino, Genova, Italy; ^5^Dipartimento di Neuroscienze, Oftalmologia, Genetica, Riabilitazione e Scienze Materno-infantili (DiNOGMI), Università di Genova, Genova, Italy; ^6^Alzheimer’s Disease Rehabilitation Unit, IRCCS Istituto Centro San Giovanni di Dio Fatebenefratelli, Brescia, Italy; ^7^Department of Neurosciences, University of Padua, Padova, Italy; ^8^Non-Invasive Brain Stimulation Unit/Department of Behavioral and Clinical Neurology, Santa Lucia Foundation IRCCS, Rome, Italy; ^9^Stroke Unit, Department of Neuroscience, Tor Vergata Policlinic, Rome, Italy; ^10^Department of Neuroscience Imaging and Clinical Sciences and CESI, University “G. D’Annunzio” of Chieti-Pescara, Chieti, Italy; ^11^Department of Motor Sciences and Healthiness, University of Naples Parthenope, Naples, Italy; ^12^Oasi Research Institute – IRCCS, Troina, Italy; ^13^Department of Neuroscience, Mental Health and Sensory Organs, Sapienza University of Rome, Rome, Italy; ^14^Department of Biophysics, School of Medicine, Istanbul Medipol University, Istanbul, Türkiye; ^15^Research Institute for Health Sciences and Technologies (SABITA), Istanbul Medipol University, Istanbul, Türkiye; ^16^Department of Neurology, Dokuz Eylül University Medical School, Izmir, Türkiye; ^17^Faculty of Medicine, Izmir University of Economics, Izmir, Türkiye; ^18^Institute for Research and Medical Care, IRCCS San Raffaele Roma, Rome, Italy; ^19^Telematic University San Raffaele, Rome, Italy; ^20^Department of Medicine and Aging Sciences, University G. D’Annunzio of Chieti-Pescara, Chieti, Italy; ^21^San Raffaele of Cassino, Cassino, Italy

**Keywords:** resting-state eyes closed electroencephalographic (rseeg) rhythms, alzheimer’s disease with dementia (add), interdependencies of rseeg rhythms, linear lagged connectivity, graph theory, hub topology

## Abstract

**Introduction:**

Graph theory models a network by its nodes (the fundamental unit by which graphs are formed) and connections. ‘Degree’ hubs reflect node centrality (the connection rate), while ‘connector’ hubs are those linked to several clusters of nodes (mainly long-range connections).

**Methods:**

Here, we compared hubs modeled from measures of interdependencies of between-electrode resting-state eyes-closed electroencephalography (rsEEG) rhythms in normal elderly (Nold) and Alzheimer’s disease dementia (ADD) participants. At least 5 min of rsEEG was recorded and analyzed. As ADD is considered a ‘network disease’ and is typically associated with abnormal rsEEG delta (<4 Hz) and alpha rhythms (8–12 Hz) over associative posterior areas, we tested the hypothesis of abnormal posterior hubs from measures of interdependencies of rsEEG rhythms from delta to gamma bands (2–40 Hz) using eLORETA bivariate and multivariate-directional techniques in ADD participants versus Nold participants. Three different definitions of ‘connector’ hub were used.

**Results:**

Convergent results showed that in both the Nold and ADD groups there were significant parietal ‘degree’ and ‘connector’ hubs derived from alpha rhythms. These hubs had a prominent outward ‘directionality’ in the two groups, but that ‘directionality’ was lower in ADD participants than in Nold participants.

**Discussion:**

In conclusion, independent methodologies and hub definitions suggest that ADD patients may be characterized by low outward ‘directionality’ of partially preserved parietal ‘degree’ and ‘connector’ hubs derived from rsEEG alpha rhythms.

## 1. Introduction

Alzheimer’s disease (AD) is the most prevalent neurodegenerative disorder in the elderly and causes cognitive deficits (e.g., episodic and working memory, executive functions, visuospatial abilities, language, etc.) and disabilities in activities of daily living progressively (i.e., loss of autonomy) belonging to dementia as a clinical syndrome (Tahami Monfared et al., 2022). It is provoked by the abnormal accumulation in the brain of Ab-42 and tau proteins, so the neurobiological *in vivo* diagnosis can be made using techniques that measure that accumulation, such as analysis of cerebrospinal fluid and positron emission tomography (Jack et al., 2019).

AD is considered a pathology affecting functional brain connectivity (Teipel et al., 2016). In this area of research, previous structural and resting-state functional magnetic resonance imaging (sMRI and rs-fMRI) studies showed colocalized abnormalities in both interhemispheric and intrahemispheric cortical connectivity in ADD patients compared with healthy elderly people (Nold) with unimpaired cognition (Delbeuck et al., 2003; Busche and Konnerth, 2016; Nakamura et al., 2017). Thanks to the high spatial resolution of MRI techniques (i.e., millimeters), those abnormalities were mainly localized as follows: (1) in the posterior parietal (precuneus) and cingulate cortices of the cortical default mode network (DMN; Bokde et al., 2006; Sorg et al., 2007; Brier et al., 2012; Wang et al., 2013, 2015; Joo et al., 2016; Eyler et al., 2019; Talwar et al., 2021; Zhang et al., 2021); (2) in the occipital and inferior parietal gyrus (Wang et al., 2021); and (3) in the medial temporal lobe and other nodes of the limbic system (Talwar et al., 2021).

Another significant contribution made by the sMRI and rs-fMRI studies, together with other brain research techniques, was to unveil the abnormal topological organization underlying the above alterations in the functional brain connectivity observed in ADD patients [see reviews by Reijneveld et al. (2007), Xie and He (2012), Stam (2014)]. In this topological organization, a cortical neural network can be formally represented by a ‘graph’ constituted of ‘nodes’ interconnected by ‘edges’. Notably, the topology of ‘nodes’ and ‘graphs’ globally reflects the following properties of a network: (1) near cortical nodes can be highly interconnected to each other forming ‘clusters’, and the nodes with more edges may have a prominent central role and underpin the **modularity** and **segregation** of the information within a network; (2) a few cortical nodes, the ‘hubs’, can ensure long-range interconnections between ‘clusters’ and may reduce the path length between far nodes and underpin the **integration** of the information within a network; (3) the ‘degree centrality’ or ‘nodal degree’ can define the importance of the hub, the ‘hub centrality’, in the information transmission within a brain network; (4) a hub can be classified as ‘connector’, connecting several different network modules, or ‘provincial’, mostly connecting nodes in the same network module as measured by the hub ‘participation coefficient’; (5) the number of the shortest paths that pass through a cortical node defines the node importance, the ‘betweenness centrality’, in the information transmission within a brain network; (6) a few highly connected cortical nodes may show dense interconnections with each other and form a sort of ‘rich club’ structure with a particular importance in the network information processing; (7) the topological distance between nodes, i.e., the mean number of edges to connect them, the ‘global efficiency’, is inversely related to effective parallel information transfer and integrated processing; and (8) an optimal balance between the network modularity (segregation) and integration of the nodes defines the so-called ‘small worldness’ structure, which is a favorable for information processing and shows **resilience** to insults impairing cortical nodes (Bullmore and Sporns, 2009, 2012; He and Evans, 2010; van den Heuvel and Sporns, 2011; Sporns, 2013; Wang et al., 2015; Liao et al., 2017).

Previous rs-fMRI studies also showed that compared with Nold people, ADD patients were characterized by decreased network segregation, as revealed by lower clustering/modular structure of the network graphs (Supekar et al., 2008; Chen et al., 2013) and higher network integration structure, as revealed by lower characteristic path length among the cortical nodes (Sanz-Arigita et al., 2010). Furthermore, prodromal ADD patients with mild cognitive impairment (ADMCI) compared with controls showed a higher global ‘clustering coefficient’, while ADD patients presented a higher hub ‘participation coefficient’ in the inferior parietal cortex, prefrontal cortex, precuneus, and somatomotor cortex (Ng et al., 2021). By contrast, diffusion MRI showed the following opposite picture in ADD patients over Nold persons: (1) lower network segregation, as revealed by a higher ‘clustering coefficient’ (Yao et al., 2010; Daianu et al., 2013); (2) lower efficiency of the network structure in relation to memory and executive performances (Lo et al., 2010; Reijmer et al., 2013); and (3) lower network integration, as revealed by higher characteristic ‘path length’ (Lo et al., 2010; Yao et al., 2010).

It should be remarked that the rs-fMRI has a low temporal resolution of about 1 s, which is insufficient to investigate the interdependency between the emerging activity of neural brain populations at frequencies higher than 0.5 Hz. Therefore, electroencephalographic (EEG) techniques were used to explore that interdependency at a larger frequency spectrum, as they have a high temporal resolution of <1 ms, despite a moderate spatial resolution of centimeters (de Haan et al., 2012). Previous EEG studies showed abnormalities in several measures of the interrelatedness of rsEEG rhythms at electrode or source pairs. Compared with Nold persons, ADD patients presented lower ‘spectral coherence’ at alpha (8–12 Hz) and beta (13–20 Hz) rhythms, especially at temporo-parieto-occipital and fronto-parietooccipital electrode pairs; notably, ‘spectral coherence’ is the most popular linear measure of the interrelatedness of rsEEG activity (Leuchter et al., 1992; Dunkin et al., 1994; Locatelli et al., 1998; Jelic et al., 2000; Adler et al., 2003). Similarly, ADD and ADMCI patients exhibited lower interrelatedness of temporo-parieto-occipital and/or fronto-parietooccipital rsEEG alpha rhythms, as revealed by the following procedures: the ‘phase lag index’, a spectral measure of the phase difference distribution asymmetry (Stam et al., 2007b; Yu et al., 2016; Peraza et al., 2018), ‘synchronization likelihood’, a measure sensitive to both linear and non-linear interrelatedness of rsEEG activity (Babiloni et al., 2004b, 2006b), and ‘linear lagged connectivity’, a measure of the interrelatedness of rsEEG activity without the zero-lag component sensitive to the head volume conduction effects (Babiloni et al., 2018, 2019).

The above rsEEG findings were confirmed and extended by measures reflecting the directionality of the interrelatedness of rsEEG activity from one electrode/source to another, such as the ‘directed transfer function’ derived from Granger causality and autoregressive methods (Blinowska, 2011; Blinowska et al., 2017). ADD and ADMCI patients exhibited lower interrelatedness of the temporo-parieto-occipital and/or fronto-parietooccipital rsEEG alpha rhythms, as revealed by “directed transfer function” (Dauwels et al., 2009, 2010). Furthermore, there was a reduced prominence of the interrelatedness from parietal to frontal electrodes at the alpha and beta (13–35 Hz) rhythms (Babiloni et al., 2008, 2009a,b; Blinowska et al., 2017).

Concerning the rsEEG delta (<4 Hz) and/or theta (4–7 Hz) rhythms, most of the studies showed higher measures of the interrelatedness of topographically widespread rsEEG activity, intrahemispherically and inter-hemispherically; those measures were derived from the ‘spectral coherence’, ‘directed transfer function’, and ‘linear lagged connectivity’ (Babiloni et al., 2008, 2009a,b, 2010, 2018, 2019; Sankari et al., 2011; Canuet et al., 2012; Yu et al., 2016; Blinowska et al., 2017), with some exceptions (Knott et al., 2000; Adler et al., 2003).

Previous rsEEG studies also revealed the abnormal network topology of the interrelatedness of rsEEG rhythms at electrode/source pairs in ADD patients. Compared with Nold people, ADD and ADMCI patients showed a more random topology of the interrelatedness of rsEEG rhythms at an electrode or source pairs, possibly due to reduced ‘small worldness’ properties of brain networks (Reijneveld et al., 2007; Stam et al., 2007a; de Haan et al., 2009; Frantzidis et al., 2014; Vecchio et al., 2014, 2016; Hallett et al., 2020). This general effect was reported at the delta, alpha, and beta rhythms on the whole scalp (Stam et al., 2007a; de Haan et al., 2009; Vecchio et al., 2014, 2016) and in AD-vulnerable regions, such as the frontal and parietal regions (Frantzidis et al., 2014).

Moreover, beyond the ‘small worldness’ property, ADD patients were characterized by abnormalities in the following graph network indexes: (1) a shift of the ‘betweenness centrality’ center of mass from posterior to anterior alpha rhythms in relation to disease severity, as revealed by the ‘phase lag index’ (Engels et al., 2015); (2) a parietal and occipital loss of the network organization from theta and alpha rhythms, as revealed by the ‘phase lag index’ (Yu et al., 2016); (3) hub rearrangement and functioning at different rsEEG frequency bands, as revealed by several interrelatedness measures (Stam et al., 2007a; De Haan et al., 2009; Frantzidis et al., 2014; Engels et al., 2015; Song et al., 2019; Das and Puthankattil, 2022); (4) lower ‘global efficiency’, increased ‘local efficiency’, and lower resilience of cortical networks from the rsEEG alpha and beta rhythms, as revealed by the Granger ‘directed transfer function’ (Afshari and Jalili, 2017); and (5) reduced graph ‘local and global efficiency’ values from lower inward and outward directions of the interrelatedness derived from the whole-band rsEEG activity by another Granger measure based on a conditional multivariate vector autoregression model. Notably, the maximum abnormalities of the ‘hub degree’ were observed at parietal electrodes (Franciotti et al., 2019), whereas no changes in the global network organization from the whole-band rsEEG activity were found by ‘mutual information’ measures of that interrelatedness (Franciotti et al., 2022).

Considering the above rsEEG findings, both ADD and ADMCI patients showed reduced efficient information exchange in the cortical neural networks, as revealed by their more random topology. However, no previous study in those patients focused on the integrity of the parietal hubs derived from the rsEEG alpha rhythms, although it is well known that ADD patients show the following significant abnormalities: (1) impairment in the parietal nodes of the cortical DMN (Bokde et al., 2006; Sorg et al., 2007; Brier et al., 2012; Wang et al., 2015; Joo et al., 2016; Eyler et al., 2019; Talwar et al., 2021; Zhang et al., 2021); (2) reduced interrelatedness of the parietal rsEEG alpha rhythms electrode or source pairs (Leuchter et al., 1992; Locatelli et al., 1998; Jelic et al., 2000; Babiloni et al., 2004a, 2006a, 2018, 2019; Stam et al., 2007b; Yu et al., 2016; Peraza et al., 2018); and (3) reduced power density of the occipital and parietal rsEEG alpha rhythms (reviewed by Babiloni et al., 2021).

In ADD patients, the abnormal reduction in rsEEG alpha rhythms may be related to disorders in the regulation of quiet vigilance. This functional interpretation is based on, among others, the following findings: (1) in healthy volunteers, posterior (eyes closed) rsEEG alpha rhythms were modulated in amplitude after transcranial magnetic stimulations over angular gyrus, a core region of the DMN, but not over control regions of the dorsal attention network (Capotosto et al., 2012); (2) those rsEEG alpha rhythms also reduced in amplitude 1 min before the onset of sleep stage 1 (Morikawa et al., 2002); (3) furthermore, they decreased in amplitude and theta rhythms increased in amplitude during the transition from quiet vigilance to drowsiness, behaviorally tested by both EEG spectral measures and reaction time and decision making to auditory stimuli (Jagannathan et al., 2018, 2022); and (4) moreover, a night of sleep deprivation reduced the posterior rsEEG alpha rhythms in healthy volunteers and visual attention performances (placebo condition), whereas an acute dose of an amphetamine (experimental condition) after sleep deprivation recovered both the posterior EEG alpha rhythms and those performances (Del Percio et al., 2019).

To fill the above literature gap, the present study explored the integrity of the parietal graph-based hubs derived from the rsEEG alpha rhythms in mild-to-moderate ADD patients compared with Nold people. In the present study, all methods for estimating the directional (isolated lagged effective coherence, iCoh) and non-directional (linear lagged connectivity, LLC) interrelatedness of the rsEEG activity at electrode pairs are implemented in the freeware platform called eLORETA.^1^ Along the same line, the methods for computing the Graph Theory indexes are implemented in the freeware platform called GraphVar.^2^ These methods were chosen to (1) use different mathematical approaches to measure that interrelatedness, (2) compare the results, (3) promote open science, and (4) allow easier cross-validation of the present results in the future. Notably, we did not want to provide a methodological standard for applying graph theory analysis. Rather, we provided a proof of concept of how the results may be affected by different thresholds and criteria.

## 2. Materials and methods

### 2.1. Subjects

For the present study, we used the clinical and rsEEG data of 40 ADD patients and 40 control Nold people carefully matched for age, gender, and education and enrolled by clinical units of our Consortium (data from three ADD patients were irremediable artifacts or relevant missing data, so were not considered further). The Local institutional Ethics Committees approved the present study. All experiments were performed with the informed and overt consent of each participant or caregiver, in line with the Code of Ethics of the World Medical Association (Declaration of Helsinki) and the standards established by the local Institutional Review Board.

Table 1 summarizes the most relevant demographic (i.e., age, gender, and education) and clinical (i.e., MMSE score) features of the Nold and ADD participants. Furthermore, it shows the results of the statistical comparisons (*p* < 0.05) of age (*t*-test), gender (Fisher test), education (*t*-test), and MMSE score (Mann–Whitney *U* test) between the two groups. As expected, a statistically significant difference was found for the MMSE score (*p* < 0.001), indicating a higher score in the Nold group than the ADD group. No difference was found for the age, gender, and education between the two groups (*p* > 0.05 uncorrected).

**Table 1:** Demographic, clinical, neuropsychological, and neurophysiological characteristics of the normal elderly (Nold) subject and Alzheimer’s Disease with Dementia (ADD) patients enrolled in the present study.

Group	*N*	Age (± SEM)	Education (± SEM)	Gender (M/F)	MMSE (± SEM)	TF (± SEM)	IAF (± SEM)
Nold	40	73.8 (± 1.0)	9.1 (± 0.5)	20/20	28.6 (± 0.2)	5.7 (± 0.1)	9.0 (± 0.2)
ADD	37	73.7 (± 1.0)	8.7 (± 0.8)	17/20	18.7 (± 0.6)	5.3 (± 0.2)	8.6 (± 0.3)
Statistical comparisons	–	t test, n.s.	t test, n.s.	–	Mann–Whitney, *p* < 0.0001	t test, n.s.	t test, n.s.

SEM, standard error of the mean; MMS, Mini-Mental State Examination; TF, transition frequency; IAF, individual alpha frequency; n.s., not significant.

In Table 1, the mean values of TF and IAF for the Nold and ADD groups, together with the results of the statistical comparisons between them (*t*-test), are also reported. No statistically significant differences were observed for TF and IAF values (*p* > 0.05 uncorrected).

### 2.2. Diagnostic criteria

In all clinical units, probable ADD was diagnosed based on the criteria of the Diagnostic and Statistical Manual of Mental Disorders, fourth edition (DSM-IV-TR; American Psychiatric Association), and the National Institute of Neurological Disorders and Stroke-Alzheimer Disease and Related Disorders (NINCDS-ADRDA) working group (McKhann et al., 1984, 2011). Diagnostic criteria refer to the time period when diagnoses were performed. All ADD individuals underwent medical, neuropsychological, neurological, psychiatric, and neuroimaging evaluations, according to standard procedures at each center and based on the expertise of each clinician. The procedures followed by all clinical units included the Instrumental Activities of Daily Living scale (IADL; Lawton and Brody, 1969), the Mini-Mental State Examination (MMSE; Folstein et al., 1975), the Clinical Dementia Rating scale (CDR; Hughes et al., 1982), the Geriatric Depression Scale (GDS; Yesavage et al., 1982), and the Hachinski Ischemic Score scale (HIS; Rosen et al., 1980).

Inclusion criteria included the clinical diagnosis of AD based on the above procedures and the determination of a worsening episodic memory in the last 6 months, thus referring to patients with typical ADD clinical presentation. According to the Alzheimer’s Disease Neuroimaging Initiative (ADNI),^3^ the MMSE score had to be 24 or lower. Additionally, inclusion criteria included the visual analysis of structural T1-weighted magnetic resonance images (MRIs) by local radiologists; those images had to be compatible with ADD diagnosis (Albert et al., 2011). Cognitive deficits were assessed by standard neuropsychological tests in the domains of episodic memory, language, executive function/attention, and visuoconstruction abilities (local normative reference thresholds). Only some of the patients received the CERAD-plus battery. In general, the tests assessing episodic memory included the delayed recall of Rey figures (Rey, 1959) and/or the delayed recall of a story (Spinnler and Tognoni, 1987). The tests assessing language included the 1-min verbal fluency for letters, fruits, animals, or car trades (Novelli et al., 1986), and/or the Token test (Spinnler and Tognoni, 1987). The tests assessing executive function and attention included the Trail Making Test Part A and B (Reitan, 1958). Finally, the tests assessing visuoconstruction abilities included the copy of Rey figures (Rey, 1959). This inhomogeneity derived from the retrospective nature of the study, with data collected during a clinical routine at each center.

Exclusion criteria included major neuropsychiatric disorders and other types or causes of dementia, such as frontotemporal dementia (Rascovsky et al., 2011), vascular dementia diagnosed based on the National Institute of Neurological Disorders and Stroke and Association Internationale pour la Recherché et l’Enseignement en Neurosciences (NINDS-AIREN) working group (Gorelick et al., 2011), Parkinson disease (Gelb et al., 1999), dementia with Lewy Bodies (McKeith et al., 2005), metabolic syndrome, nutritional deficits, tumors, epilepsy, etc. Exclusion criteria also included visual analysis of structural T2-weighted MRIs by local radiologists to exclude major cerebrovascular lesions, as well as the chronic use of psychoactive drugs except for acetylcholinesterase inhibitors (all patients chronically took them) and/or NMDA receptor antagonists.

The Nold participants received a cognitive, physical, and neurological examination to exclude the presence of cognitive deficits and psychiatric disorders. According to ADNI, the MMSE score had to be 27 or higher. Additionally, all Nold participants had a GDS score lower than the threshold of 5 (no depression) or were verified as not having depression after an interview with a physician or clinical psychologist. Those affected by chronic systemic illnesses (e.g., diabetes mellitus) were excluded, as well as participants receiving chronic psychoactive drugs. Assessed Nold people were also excluded if they had, currently or historically, neurological or psychiatric diseases and drug or alcohol abuse issues.

In ADD and Nold participants, pharmacological administration (when planned) of routine drugs was postponed until after the rsEEG recordings and performed in hospital settings in the morning. Although this procedure did not guarantee a full washout of the drugs, it synchronized the timing of drug administration. Longer periods of suspension would not have been valid for obvious ethical reasons.

### 2.3. Resting state eyes-closed electroencephalographic recordings

In all clinical units, the Nold and ADD participants were kindly asked to stay relaxed with their eyes closed during the experiments. They were also kindly asked not to move or talk and keep their mind wandering without focused mentalization. During the experimental recordings, the researchers controlled for the subject’s behavioral condition and ongoing rsEEG traces (specifically the amplitude of alpha waves on posterior regions and the onset of slow-wave activity in frontal regions), helping the participants to keep an adequate level of vigilance (i.e., avoiding drowsiness and sleep onset). These alarms were annotated in the protocol for the preliminary rsEEG data analysis phase. The above instructions and procedures were similar in all clinical units even if the respective protocols were not identical.

**Figure 1:**
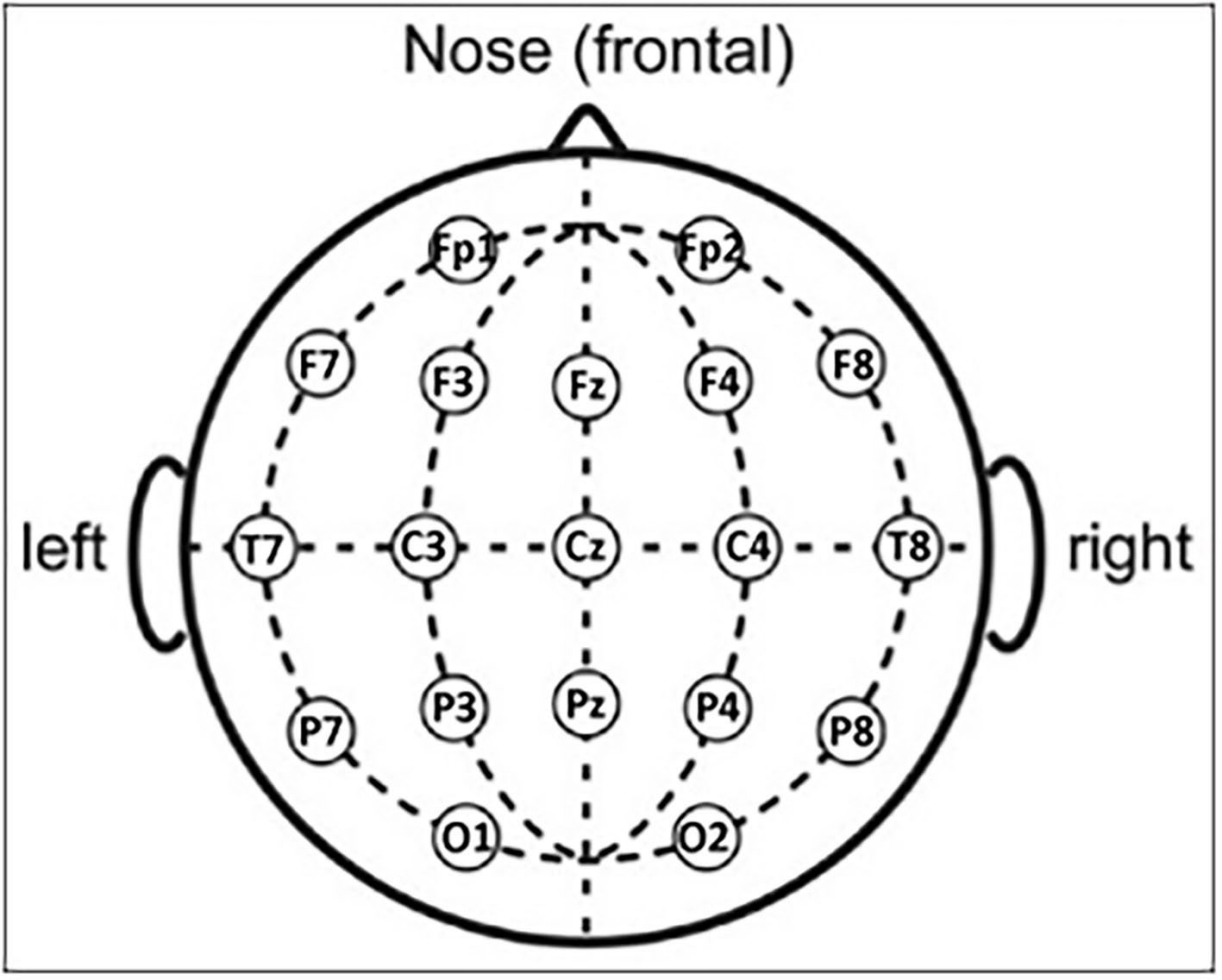
Scalp electrode positioning of the 19 electrodes according to the international standard 10–20.

At least 5 min of electrophysiological data were recorded by professional digital EEG systems authorized for clinical applications (i.e., EB-Neuro Be-light, Micromed, Brain Product, etc.). For this purpose, 19 exploring scalp electrodes were placed according to the 10–20 montage system (i.e., Fp1, Fp2, F7, F3, Fz, F4, F8, T7, C3, Cz, C4, T8, P7, P3, Pz, P4, P8, O1, and O2; Figure 1).

The ground electrodes were placed in the posterior midline, while the reference electrodes were located in different positions across participating clinical units (i.e., linked earlobes, mastoids, vertex, etc.), in line with local standard protocols and clinical trials. During the rsEEG recordings, scalp electrode impedances were kept below 5 KOhm. The rsEEG recordings were performed using 128 Hz or a higher sampling rate (i.e., 128–1,024 Hz) with an adequate antialiasing band pass between 0.01 Hz and 60–100 Hz.

In addition to the rsEEG recording, bipolar vertical and horizontal electrooculographic (EOG) signals and one-channel electrocardiographic (ECG) signals were also acquired using the same sampling frequency adopted for recording the rsEEG data (128–1,024 Hz). Consequently, rsEEG, EOG, and ECG signals had the same sampling rate, so EOG and ECG signals could be used for artifact detection and their off-line correction when adequate.

As mentioned above, some rsEEG datasets were recorded using a relatively low sampling frequency of 128 Hz (i.e., 6 out of 40 rsEEG datasets collected for the Nold group and 4 out of 37 rsEEG datasets collected for the ADD group). It should be remarked that such a sampling frequency is suboptimal for an ideal reconstruction of rsEEG signal beyond 40 Hz without aliasing. Ideally, a factor of 3–4 between the low-band pass limit and the rsEEG sampling frequency should be set.

### 2.4. Preliminary rsEEG data analysis

Data analysis was centrally performed by the group located at the Department of Physiology and Pharmacology ‘Erspamer’ of Sapienza University of Rome, Italy. In the preliminary analysis, the rsEEG data were split into 2-s epochs and analyzed off-line. This segmentation allowed the use of standard toolboxes for the spectral analysis of rsEEG activity, such as fast Fourier transform (FFT) implemented in the official eLORETA platform. This analysis assumes the stationarity of rsEEG activity. Furthermore, it allowed for the minimization of the rejection of rsEEG data for artifactual activity. The use of those procedures allowed a better understanding of the present results in light of previous reference evidence of the PDWAVES Consortium (Babiloni et al., 2015, 2016; Lizio et al., 2016; more information can be found at www.pdwaves.eu), but it implied the focus on the linear components of rsEEG signals.

Two independent researchers (GN and RL) performed a visual analysis of EOG and rsEEG data blind to the clinical diagnosis associated with the electrophysiological datasets. They rejected those with artifacts due to instruments, electronic noise, head–neck movements, and face muscle tension. They also rejected rsEEG epochs with amplitude values exceeding 100 μV. Particular attention was given to the contamination of saccades and blinking on electrophysiological data recorded by frontal (i.e., F7, F3, Fz, F4, and F8) and frontopolar (Fp1 and Fp2) electrodes. This specific exam was based on the comparison of EOG and rsEEG traces. The rsEEG epochs with artifacts marked as eye movements and blinking were provided as inputs to a software toolbox based on an autoregressive model for their possible correction (MATLAB 6.5, MathWorks Inc.). Technical details and performances of this procedure have been reported elsewhere (Moretti et al., 2003) and validated in several previous studies by the present research group (Babiloni et al., 2004a, 2006a, 2008). Of note, the outcome of this procedure was visually revised by the two researchers (G. N. and R. L.). All Nold and ADD datasets showed less than 25% of artifact-free rsEEG epochs, without significant differences between the Nold and ADD groups (*t*-test, *p* > 0.05, two tails). More specifically, the total number of artifact-free epochs was as follows: 135 ± 11 (SE) epochs for the Nold group and 115 ± 8 (SE) epochs for the ADD group, with a total duration spanning between 3.5 and 4.5 min, respectively.

To harmonize rsEEG data recorded using different reference electrodes and sampling frequency rates, artifact-free rsEEG epochs were off-line frequency-band passed at 0.1–45 Hz and downsampled, when appropriate, to make the sampling rate of all artifact-free rsEEG datasets in the Nold and ADD participants equal to 128 Hz. For the sake of harmonization of all datasets, the recorded rsEEG data were re-referenced to the common average reference.

### 2.5. The spectral analysis of rsEEG epochs

A standard digital FFT-based analysis (Welch technique, Hanning windowing function, no phase shift) computed the power density of scalp rsEEG rhythms (0.5 Hz of frequency resolution). As mentioned above, only rsEEG epochs free from artifacts were used.

The EEG frequency bands of interest were individually identified based on the following frequency landmarks: the transition frequency (TF) and the individual alpha frequency (IAF) peak (Klimesch, 1999). In the EEG power density spectrum, the TF marks the transition frequency between the theta and alpha bands, defined as the minimum of the rsEEG power density between 3 and 8 Hz (between the delta and the alpha power peak). The IAF is defined as the maximum power density peak between 6 and 14 Hz. These frequency landmarks were previously well described by Dr. Wolfgang Klimesch (Klimesch, 1996, 1999; Klimesch et al., 1998). Specifically, the TF and IAF were measured on averaged rsEEG power density spectra at parietal and occipital electrodes.

The TF and IAF were computed for each subject involved in the study. Based on the TF and IAF, we estimated the individual delta, theta, and alpha bands as follows: delta from TF −4 Hz to TF −2 Hz, theta from TF −2 Hz to TF, low-frequency alpha (alpha 1 and alpha 2) from TF to IAF, and high-frequency alpha (or alpha 3) from IAF to IAF + 2 Hz. Specifically, the individual alpha 1 and alpha 2 bands were computed as follows: alpha 1 from TF to the frequency midpoint of the TF-IAF range and alpha 2 from that midpoint to IAF.

The other bands were defined based on the standard fixed frequency ranges used in the reference study series (reviewed by Babiloni et al., 2021): beta 1 from 14 to 20 Hz, beta 2 from 20 to 30 Hz, and gamma from 30 to 40 Hz. See Supplementary Figure 1 in the Supplementary materials for the graphical representation of the above-mentioned frequency bands.

Of note, important aspects of the procedure were as follows:

1. The alpha band was divided into sub-bands because, in the rsEEG data, dominant low-frequency alpha rhythms (alpha 1 and alpha 2) may denote the synchronization of diffuse neural networks regulating the fluctuation of the subject’s global awake and conscious states, while high-frequency alpha rhythms (alpha 3) may denote the synchronization of more selective neural networks specialized in the processing of modal specific or semantic information (Pfurtscheller and Klimesch, 1992; Klimesch, 1999). When the subject is engaged in sensorimotor or cognitive tasks, alpha and low-frequency beta (beta 1) rhythms do reduce in power (i.e., desynchronization or blocking) and are replaced by fast EEG oscillations at high-frequency beta (beta 2) and gamma rhythms (Pfurtscheller and Klimesch, 1992).
2. We considered individual delta, theta, and alpha frequency bands because a clinical group may be characterized by a mean slowing in the peak frequency of the alpha power density without any substantial change in the magnitude of the power density. In that specific case, the use of fixed frequency bands would result in a statistical effect erroneously showing alpha power density values lower in the clinical group than in the control group. In some specific cases, the groups of AD patients and control participants may not show statistically significant differences in the mean values of TF and IAF. Nevertheless, we used those values as a research model to allow the identification of delta, theta, low-frequency alpha bands, and high-frequency alpha bands on an individual basis to ensure the spectral measures were accurate within those bands, in line with our reference rsEEG studies performed in patients with AD and related neurodegenerative disorders (Babiloni et al., 2017, 2018, 2019, 2020).
3. Fixed frequency ranges were used for the beta and gamma bands because the individual beta and gamma frequency peaks were only evident in a few subjects (<10%).
4. We selected the beginning of the beta frequency range at 14 Hz to avoid overlapping between individual alpha and fixed beta frequency ranges (i.e., the individual alpha frequency band ranged from TF to 14 Hz with an IAF of 12 Hz).

During rsEEG recording, very careful attention was paid to the amplitude of alpha rhythms on posterior regions and the abnormal slow wave on frontal regions. Overall, specific spectral features should be respected, namely:

1. The physiological decrease of the EEG power density after the IAFp as a function of the increase of the frequencies in the range of 1–40 Hz (related to residual muscular activity);
2. The absence of an offset of power density across all frequencies at some scalp electrodes (especially visible as big differences in gamma rsEEG power density among the ROI);
3. The absence of several peaks of high-power density in the range of 1–40 Hz; and.
4. Visible IAFp in the range between 6 Hz and 14 Hz, especially on posterior regions.

We carefully checked the presence of IAFp in the present cohort of AD patients, as in a mild-to-moderate dementia stage, AD neuropathology should not impair the neurophysiological synchronizing mechanism inducing a total disruption of IAFp. If an IAFp was not clearly present, we attributed the cause to substantial artifacts rather than AD neuropathology.

### 2.6. Estimation of linear lagged connectivity (LLC) and isolated lagged effective coherence (iCoh)

As mentioned above, LLC and iCoh are two complementary and mathematically independent approaches available at the freeware platform called LORETA (see technical details at https://www.uzh.ch/keyinst/loreta; Pascual-Marqui, 2007) for measuring the interrelatedness of rsEEG activity at electrode (source) pairs. Comparing the results with two techniques probed the intrinsic variability of this kind of readout and allowed us to select and discuss the one that was most consistent.

LLC belongs to the popular bivariate techniques that compute the non-directional interrelatedness of rsEEG activity at electrode pairs (e.g., spectral coherence, phase lag index, synchronization likelihood, etc.) without considering the interrelatedness of rsEEG activity across the other electrode (source) pairs. It has the conceptual advantage of not considering the interrelatedness of the rsEEG activity at the zero-lag phase, which may be affected by the instantaneous spread of the electric field to the well-known head volume conduction effects (Pascual-Marqui et al., 2011).

By contrast, iCoh belongs to a group of techniques based on an autoregressive model that computes the directional interrelatedness of rsEEG activity at electrode pairs (e.g., spectral coherence, phase lag index, synchronization likelihood, etc.), removing the linear component of the interrelatedness of rsEEG activity across the other electrode (source) pairs. It has the conceptual advantage of being multivariate (as opposed to bivariate) and exploring the directionality of that interrelatedness (Pascual-Marqui et al., 2014).

Using the iCoh procedure, we obtained not only a ‘directional’ measure of the interdependencies of rsEEG rhythms at electrode pairs but also a ‘non-directional’ measure. The directional measure was computed as the absolute difference of the iCoh values between the two ‘directions’, while the ‘non-directional’ measure was obtained by the mean of the two ‘directional’ values. The latter measure allowed cross-validation of (‘non-directional’) LLC measures.

For each participant, LLC, mean iCoh between the two ‘directions’, and the absolute difference of the iCoh values for the two ‘directions’ were calculated at each frequency bin between 0.5 and 45 Hz (matrix of 19 rows × 19 columns). LLC and iCoh values within the frequency bands individually identified based on the TF and IAF landmarks were averaged to obtain delta, theta, alpha 1, alpha 2, and alpha 3 bands. LLC and iCoh values for beta 1, beta 2, and gamma LLC were based on fixed frequency bands, as mentioned above.

To reduce statistical comparisons, we averaged the LLC or iCoh values calculated between scalp electrode pairs for regions of Interests (ROI). Specifically, we considered frontal, central, parietal, temporal, and occipital ROI. For each frequency band, LLC or iCoh values for interhemispheric comparisons were calculated as follows: (1) frontal ROI, mean values of Fp1-Fp2, F3-F4, and F7-F8 electrodes; (2) central ROI, the values of C3-C4 electrodes; (3) parietal ROI, the values of P3-P4 electrodes; (4) temporal ROI, mean values of T7-T8 and P7-P8 electrodes; and (5) occipital ROI, the values of O1-O2 electrodes.

Similarly, for each frequency band, LLC or iCoh values for intrahemispheric comparisons were calculated as follows: (1) left frontal ROI, mean values of electrode pairs between Fp1, F3, and F7 electrodes and all the left hemispheric electrodes; (2) right frontal ROI, mean values of electrode pairs between Fp2, F4, and F8 electrodes and all the right hemispheric electrodes; (3) left central ROI, mean values of electrode pairs of the left hemi-scalp involving the C3 electrode and all the left hemispheric electrodes; (4) right central ROI, mean values of electrode pairs of the right hemi-scalp involving the C4 electrode and all the right hemispheric electrodes; (5) left parietal ROI, mean values of electrode pairs of the left hemi-scalp involving the P3 electrode and all the left hemispheric electrodes; (6) right parietal ROI, mean values of electrode pairs of the right hemi-scalp involving the P4 electrode and all the right hemispheric electrodes; (7) left temporal ROI, mean values of electrode pairs of the left hemi-scalp involving the T7 and P7 electrodes and all the left hemispheric electrodes; (8) right temporal ROI, mean values of electrode pairs of the right hemi-scalp involving the T6 and P8 electrodes and all the right hemispheric electrodes; (9) left occipital ROI, mean values of electrode pairs of the left hemi-scalp involving the O1 electrode and all the left hemispheric electrodes; and (10) right parietal ROI, mean values of electrode pairs of the right hemi-scalp involving the O2 electrode and all the right hemispheric electrodes.

### 2.7. Graph theory analysis of LLC and iCoh values

For each participant, the LLC, mean iCoh, and absolute difference of iCoh values at the frequency bands showing statistically significant differences between the ADD and Nold groups were used as input for the graph theory analysis. This analysis was performed using the GraphVar 2.0 software platform (Waller et al., 2018).

For this purpose, matrices of LLC, mean iCoh, and absolute difference of iCoh values were converted into binary matrices having ‘0’ or ‘1’ in the cells. LLC or iCoh values associated with ‘1’ were considered as ‘significant’ and considered for the computation of the graph indexes of interest in the Nold and ADD groups. Notably, we converted the LLC and iCoh matrices into binary (‘1’ and ‘0’) graphs to (1) mitigate the inclusion of ‘spurious’ interdependencies of rsEEG rhythms at electrode pairs and (2) compare graphs with the same number of those interdependencies for the Nold and ADD groups.

For the identification of the ‘significant’ values of LLC or iCoh (‘1’ in the binary matrices), two ***arbitrary percentage thresholds*** were used, namely 10% (0.1) and 20% (0.2). For each frequency band and group of participants (Nold and ADD), the threshold at 10% did set to ‘1’ the 10% of the highest values of LLC (iCoh), considering all electrode pairs, and ‘0’ for the remaining ones. This procedure was repeated for LLC, mean iCoh, and the absolute difference of iCoh values. Specifically, 10% of the highest values of LLC corresponded to 17 electrode pairs. The same number of electrode pairs was true for (‘non-directional’) mean iCoh and the absolute difference of the two ‘directional’ iCoh values.

Following the same procedure, the threshold at 20% did set to ‘1’ the 20% of the highest values of LLC (iCoh), considering all electrode pairs, and ‘0’ for the remaining ones. Specifically, 20% of the highest values of (‘non-directional’) LLC corresponded to 34 electrode pairs. Again, this procedure was repeated for LLC, mean iCoh, and the absolute difference of iCoh values. As another step of the graph theory analysis, we ***arbitrarily*** used the ***nodal degree*** (ND), ***participation coefficient*** (PC), and local ***clustering coefficient*** (CC) graph indexes to scalp electrodes as ***degree*** hubs and then differentiate them into ***provincial*** and ***connector*** hubs.

For this purpose, ND was defined as the number of links (i.e., ‘significant’ interdependencies of rsEEG rhythms at electrode pairs represented as ‘1’ in the previously mentioned binary matrices) characterizing a given node (electrode). Among nodes with a high number of links (high-degree nodes = ***degree hubs***), PC denoted the discriminant feature of their connection profile. In general, ***provincial*** hubs primarily link other nodes located within a single network region. By contrast, connector hubs predominantly link nodes located in several network regions (Sporns et al., 2007; Power et al., 2013). Here, this classification as provincial hub vs. connector hub was further confirmed by the CC index, which is a measure of the tendency of network nodes to form local clusters. High CC values mainly characterize provincial hubs rather than connector hubs.

In the present experimental context, we operationally defined ***degree*** hubs and then differentiated them into ***provincial*** and ***connector*** hubs using the following three approaches:

1. Hubs were defined according to ND and classified into connector and provincial hubs according to the PC and CC calculated at 0.1 and 0.2 graph thresholds, in line with the Franciotti and Bonanni approach.
2. Hubs were defined according to ND and classified into connector and provincial hubs according to the PC and betweenness centrality (BC) calculated at 0.1 and 0.2 graph thresholds, in line with the approach described by Cole et al. (2015).
3. Hubs were defined according to the within-module degree z-score and classified into connector and provincial hubs according to the PC calculated at 0.1 and 0.2 graph thresholds, in line with the approach described by Power et al. (2013).

Furthermore, we used the four following alternative criteria for testing the consistency of the results (they were applied for each frequency band and each group of participants):

1. According to the first criterion, ***degree hubs*** were defined as nodes (electrodes) with ND (number of links) higher than ***one standard deviation*** (SD) from the group mean of significant node links (i.e., ‘1’ in the previously mentioned binary matrices) within the network (electrode montage). ***Provincial hubs*** were then defined as degree hubs with an ND and a CC higher than one SD and PC lower than one SD from the group mean of significant node links within the network. ***Connector hubs*** were defined as degree hubs (electrodes) with an ND and a PC higher than one SD from the network mean and a CC lower than one SD from the group mean of significant node links within the network.
2. According to the second criterion, ***degree hubs*** were defined as nodes with an ND higher than the ***80th percentile*** from the group mean of significant node links within the network. ***Provincial hubs*** were then defined as degree hubs with an ND and a CC higher than the ***80th percentile*** and a PC lower than the ***20th percentile*** from the group mean of significant node links within the network. ***Connector hubs*** were defined as degree hubs with an ND and a PC higher than the ***80th percentile*** from the network mean and a CC lower than the ***20***^***th***^ ***percentile*** from the group mean of significant node links within the network.
3. According to the third criterion, ***degree hubs*** were defined as nodes with an ND higher than the ***70th percentile*** from the group mean of significant node links within the network. ***Provincial hubs*** were then defined as degree hubs with an ND and a CC higher than the ***70th percentile*** and a PC lower than the ***30th percentile*** from the group mean of significant node links within the network. ***Connector hubs*** were defined as degree hubs with an ND and a PC higher than the ***70th percentile*** from the network mean and a CC lower than the ***30th percentile*** from the group mean of significant node links within the network.
4. According to the fourth criterion, ***degree hubs*** were defined as nodes with an ND higher than ***one standard error of the mean*** (SEM) from the group mean of significant node links within the network. ***Provincial hubs*** were then defined as degree hubs with an ND and a CC higher than one SEM and a PC lower than one SEM from the group mean of significant node links within the network. ***Connector hubs*** were defined as degree hubs with an ND and a PC higher than one SEM from the network mean and a CC lower than one SEM from the group mean of significant node links within the network.

All results obtained with the above criteria are reported in detail in the tables featured in the Supplementary materials (see Results). Of note, the selection of the criteria was performed to provide an index of the result variability using different mathematical threshold definitions.

### 2.8. Directionality of degree hubs by iCoh values

To evaluate the directionality of the interdependencies of rsEEG rhythms between degree hubs (electrodes), ‘directional’ iCoh values for pairs of those hubs were calculated at each frequency bin between 0.5 and 45 Hz and for each participant of the ADD and Nold groups. Then, these iCoh values were averaged according to individual frequency bands from delta to alpha 3. To limit the statistical comparisons, the subsequent analysis was focused on individual delta, alpha 2, and alpha 3 bands, which are typically abnormal in rsEEG rhythms recorded in ADD patients (Babiloni et al., 2021). For each frequency band and group of participants, the global output (outward) value of a given degree hub was obtained averaging all output iCoh values from it to the other degree hubs. The global input (inward) value of that degree hub was obtained averaging all input iCoh values to it coming from the other degree hubs.

### 2.9. Statistical analysis

To evaluate the study hypotheses, the following statistical sessions were performed by the commercial tool STATISTICA 10 (StatSoft Inc.).^4^ As analysis of variance (ANOVA) implies that dependent variables have Gaussian distributions, we tested this feature with the LLC and iCoh values using a Kolmogorov–Smirnov test (null hypothesis of non-Gaussian distributions tested at *p* > 0.05). Both the LLC and iCoh values showed non-Gaussian distributions, so we Log10 transformed them and retested Gaussian status. Such a transformation is a popular method for transforming a skewed data distribution with all positive values, such as LLC and iCoh values, to Gaussian distributions, as required when using ANOVA. Indeed, the outcome of the procedure did approximate the distributions of LLC and iCoh values to Gaussian distributions (*p* > 0.05), allowing the use of the ANOVA model.

For the session using ANOVAs, Mauchly’s test evaluated the sphericity assumption, and degrees of freedom were corrected using the Greenhouse–Geisser procedure when appropriate (*p* < 0.05). the Duncan test was used for *post-hoc* comparisons (*p* < 0.05, corrected for multiple comparisons).

The results of the following ANOVAs were controlled by the iterative (leave-one-out) Grubbs’ test detecting for the presence of one or more outliers in the distribution of the LLC and iCoh values showing the significant effects in relation to the study hypotheses. The null hypothesis of the non-outlier status was tested at the arbitrary threshold of *p* > 0.001 to remove only values with the highest probability of being outliers.

In the first statistical session, we evaluated whether the LLC, mean iCoh, and absolute difference of iCoh interhemispheric values may differ between the ADD and Nold groups at parietal delta and alpha rhythms. To this aim, we developed three ANOVA designs with the Log10-transformed LLC, mean iCoh, and absolute difference of iCoh values as dependent variables, respectively. The factors were group (Nold, ADD; independent variable), ROI (frontal, central, parietal, temporal, and occipital), and band (delta, theta, alpha 1, alpha 2, alpha 3, beta 1, beta 2, and gamma). The confirmation of the hypothesis would require (1) a statistically significant ANOVA interaction, including the factors group, ROI, and band (*p* < 0.05), and (2) a *post-hoc* Duncan test indicating statistically significant (*p* < 0.05) differences in the LLC values at parietal delta and alpha rhythms between the Nold and ADD groups (i.e., Nold ≠ ADD, *p* < 0.05).

In the second session, we evaluated whether the LLC, mean iCoh, and absolute difference of iCoh intrahemispheric values may differ between the ADD and Nold groups at delta and alpha rhythms within the two hemispheres. To this aim, we developed three ANOVA designs with the Log10-transformed LLC, mean iCoh, and absolute difference of iCoh values as dependent variables, respectively. The factors were group (Nold, ADD; independent variable), hemisphere (left, right), ROI (frontal, central, parietal, temporal, and occipital), and band (delta, theta, alpha 1, alpha 2, alpha 3, beta 1, beta 2, and gamma). The confirmation of the hypothesis would require (1) a statistically significant ANOVA interaction, including the factors group, ROI, and band (*p* < 0.05), and (2) a *post-hoc* Duncan test indicating statistically significant (*p* < 0.05) differences in the LLC values at parietal delta and alpha rhythms between the Nold and ADD groups (i.e., Nold ≠ ADD, *p* < 0.05).

In the third session, we evaluated whether the global output and input iCoh values of parietal degree hubs (identified by the previous graph theory analysis) may differ between the ADD and Nold groups at delta and alpha rhythms. We also evaluated whether, within each group, a difference between global output and input iCoh values for degree hubs could be observed. To these aims, for each frequency band, we developed an ANOVA design with the Log10-transformed global iCoh values as a dependent variable and the group (Nold, ADD; independent variable), hub (all degree hubs), and direction (output, input) as factors. The confirmation of the hypothesis would require (1) a statistically significant ANOVA interaction, including the factors group, hub, and direction (*p* < 0.05), (2) a *post-hoc* Duncan test indicating statistically significant (*p* < 0.05) between-group differences in the parietal iCoh values at delta and alpha rhythms between the Nold and ADD groups (i.e., Nold ≠ ADD, *p* < 0.05), and (3) a *post-hoc* Duncan test indicating statistically significant (*p* < 0.05) within-group differences between the global output and input iCoh values (i.e., output ≠ input, *p* < 0.05).

## 3. Results

### 3.1. Demographic, clinical, neuropsychological, and rsEEG features in the Nold and Add groups

Table 1 summarizes the most relevant demographic (i.e., age, gender, and education) and clinical (i.e., MMSE score) features of the groups of Nold (*N* = 40) and ADD (N = 37) participants. Furthermore, it reports results of the statistical comparison (*p* < 0.05) of age (*t*-test), gender (Fisher test), education (*t*-test), and MMSE score (Mann–Whitney *U* test) between the two groups. As expected, a statistically significant difference was found for the MMSE score (*p* < 0.001), indicating a higher score in the Nold group than in the ADD group. No difference was found for age, gender, and education between the two groups (*p* > 0.05 uncorrected).

In Table 1, the mean values of TF and IAF for the Nold and ADD groups, together with the results of the statistical comparisons between them (*t*-test), are also reported. No statistically significant differences were observed for TF and IAF values (*p* > 0.05 uncorrected).

### 3.2. Interdependencies of rsEEG rhythms at parietal electrode pairs as revealed by LLC and iCoh values

Results showed a statistically significant ANOVA interaction (*F*[28, 2,100] = 2.25, *p* < 0.05) in ***interhemispheric LLC*** values among the factors group (Nold and ADD; independent variable), ROI (frontal, central, parietal, temporal, and occipital), and band (delta, theta, alpha 1, alpha 2, alpha 3, beta 1, beta 2, and gamma; Supplementary Figure 2). Compared with the ***Nold group***, the ***ADD group*** was mainly characterized by (1) lower alpha 2 and alpha 3 LLC values at parietal and temporal ROI and (2) higher delta LLC values at frontal, parietal, and occipital ROI (*p* < 0.05). No significant ANOVA effect was observed in the ***interhemispheric iCoh*** values (*p* > 0.05; see Supplementary Figures 3, 4).

Additionally, a statistically significant ANOVA interaction (*F*[28, 2,100] = 3.32, *p* < 0.05) in ***intrahemispheric (non-directional) LLC*** values among the factors group, ROI, and band (Supplementary Figure 5) was observed. Compared with the ***Nold group***, the ***ADD group*** was mainly characterized by (1) lower LLC alpha 2 and alpha 3 values at the central, parietal, and occipital ROI and (2) higher LLC delta values at frontal, parietal, and occipital ROI (*p* < 0.05).

Another statistically significant ANOVA interaction (*F*[28, 2,100] = 1.91, *p* < 0.05) was found in ***intrahemispheric (non-directional) mean iCoh*** values among the factors group, hemisphere, ROI, and band (Supplementary Figure 6). No significant *post-hoc* effect was observed in the planned tests (*p* > 0.05). There was just a trend for lower left-parietal ***iCoh*** alpha 2 and alpha 3 values in the ADD group compared with the Nold group.

Finally, a statistically significant ANOVA interaction (*F*[28, 2,100] = 2.13, *p* < 0.05) was found in the ***intrahemispheric (directional) absolute difference of iCoh values*** among the factors group, hemisphere, ROI, and band (Supplementary Figure 7). Again, no significant *post-hoc* effect was observed in the planned tests (*p* > 0.05) but there was a trend showing lower left-parietal ***iCoh*** values in the ADD group compared with the Nold group.

Table 2 reports all the results from the planned *post-hoc* tests (*p* < 0.05) for the above significant ANOVA effects. Notably, the above findings based on LLC and iCoh values were not due to outliers, as shown by Grubbs’ test with an arbitrary threshold of *p* > 0.001 (see Supplementary Figures 8, 9, respectively).

**Table 2:** *Post-hoc p*-values (Duncan test) relative to the ANOVA interaction effects on the global output and input isolated lagged effective coherence (iCoh) in the alpha 2 and alpha 3 bands.

Global alpha2 iCoh		Global alpha3 iCoh	
Statistical comparison	*p*-value	Statistical comparison	*p*-value
*Nold > ADD*	*Output P3:* 0.026011 *Output Pz:* 0.038590	*Nold > ADD*	*Output Pz:* 0.021034
*Input > Output (Nold)*	*Fp2:* 0.044984 *F3:* 0.024305 *F4:* 0.026964 *F7:* 0.038887	*Input > Output (Nold)*	*Fp2:* 0.034870 *F3:* 0.026717 *F4:* 0.027734 *F7:* 0.026067
*Input > Output (ADD)*	*Pz:* 0.013233		
*Output > Input (Nold)*	*P3:* 0.000002 *P4:* 0.000002	*Output > Input (Nold)*	*P3:* 0.000002 *P4:* 0.000003 Pz: 0.027605
*Output > Input (ADD)*	*P3:* 0.014394 *P4:* 0.000001	*Output > Input (ADD)*	*P3:* 0.007453 *P4:* 0.000005

Globally, the above LLC and iCoh findings showed that ***interdependencies of rsEEG alpha 2 and alpha 3 rhythms at parietal electrode pairs were lower in the ADD group than in the Nold group***. By contrast, results on interdependencies of rsEEG delta rhythms at scalp electrode pairs were inconsistent considering LLC and iCoh measures, so we did not use those measures for the graph hub analysis.

### 3.3. Parietal graph degree hubs from LLC and iCoh values at alpha rhythms

In the Supplementary material Tables 2–7 report detailed results about the graph degree hubs derived from LLC and iCoh alpha 2 and alpha 3 values computed in the Nold and ADD groups. As explained in the Materials and Methods section, those degree hubs were defined by the ND graph index using four different quantitative thresholds of qualification (i.e., mean + 1 SD, 80th percentile, 70th percentile, mean + 1 SEM).

**Figure 2:**
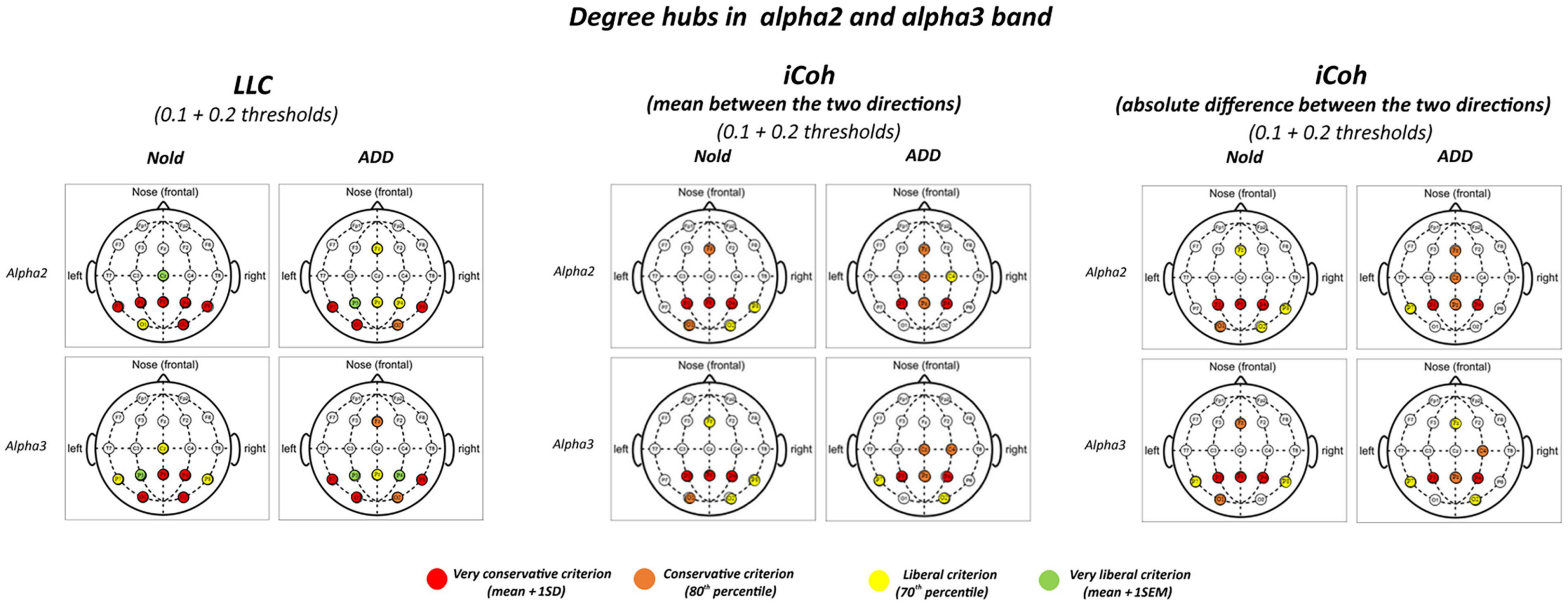
Degree hubs for the alpha 2 and alpha 3 linear lagged connectivity (LLC, left column), mean (middle column), and absolute difference isolated lagged effective coherence (iCoh, right column) values in the Nold and ADD groups defined according to nodal degree (ND) calculated at 0.1 and 0.2 graph thresholds. Colors correspond to the different criteria adopted (mean and SD, 80th percentile, 70th percentile, mean and SEM).

Figure 2 illustrates those graph degree hubs computed from LLC and iCoh alpha 2 and alpha 3 values in the Nold and ADD groups. For sake of concision, the 0.1 and 0.2 thresholds are displayed in the same figure.

Although there was a certain spread of degree hubs over the scalp at alpha 2 and alpha 3 bands, LLC and iCoh measures showed converging evidence of parietal degree hubs at these bands in both the Nold and ADD groups. Even using the most conservative criterion for the degree hub qualification (i.e., mean + 1 SD), consistent degree hubs from alpha 2 and alpha 3 rhythms were observed at parietal electrodes (i.e., P3, Pz, and P4). Notably, the iCoh values showed ***no substantial between-group differences in the topology of the parietal degree hubs at the alpha 2 and alpha 3 bands***.

### 3.4. Parietal graph connector hubs from LLC and iCoh values at alpha 2 and alpha 3 bands

In the Supplementary material Tables 8–13 report detailed results about the graph connector and provincial hubs derived from LLC and iCoh alpha 2 and alpha 3 values computed in the Nold and ADD groups. Notably, the results showed no substantial graph provincial hub in the two groups.

**Figure 3:**
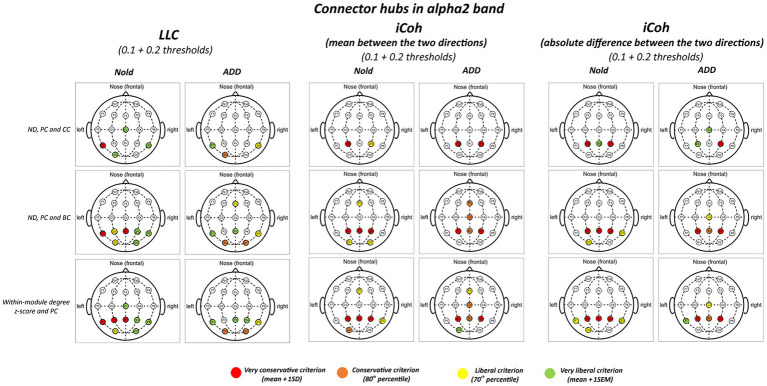
Connector and provincial hubs for the alpha 2 linear lagged connectivity (LLC, left column), mean (middle column), and absolute difference isolated lagged effective coherence (iCoh, right column) values in the Nold and ADD groups defined according to the three approaches used in the present study. Upper row: hubs were defined according to nodal degree (ND) and classified into connector and provincial hubs according to the participation coefficient (PC) and clustering coefficient (CC) calculated at 0.1 and 0.2 graph thresholds, in line with the approach described by Franciotti and Bonanni. Middle row: hubs were defined according to nodal degree (ND) and classified into connector and provincial hubs according to the participation coefficient (PC) and betweenness centrality (BC) calculated at 0.1 and 0.2 graph thresholds, in line with the approach described by Cole et al. (2015). Lower row: hubs were defined according to the within-module degree z-score and classified into connector and provincial hubs according to the participation coefficient (PC) calculated at 0.1 and 0.2 graph thresholds, in line with the approach described by Power et al. (2013). Colors correspond to the different criteria adopted (mean and SD, 80th/20th percentile, 70th/30th percentile, mean and SEM).

**Figure 4:**
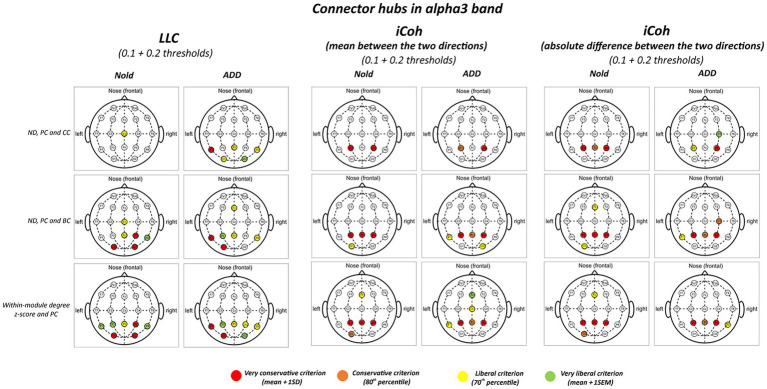
Connector and provincial hubs for the alpha 3 linear lagged connectivity (LLC, left column), mean (middle column), and absolute difference isolated lagged effective coherence (iCoh, right column) values in the Nold and ADD groups defined according to the three approaches used in the present study. Upper row: hubs were defined according to nodal degree (ND) and classified into connector and provincial hubs according to the participation coefficient (PC) and clustering coefficient (CC) calculated at 0.1 and 0.2 graph thresholds, in line with the approach described by Franciotti and Bonanni. Middle row: hubs were defined according to nodal degree (ND) and classified in connector and provincial hubs according to the participation coefficient (PC) and betweenness centrality (BC) calculated at 0.1 and 0.2 graph thresholds, in line the approach described by Cole et al. (2015). Lower row: hubs were defined according to the within-module degree z-score and classified into connector and provincial hubs according to the participation coefficient (PC) calculated at 0.1 and 0.2 graph thresholds, in line with the approach described by Power et al. (2013). Colors correspond to the different criteria adopted (mean and SD, 80th/20th percentile, 70th/30th percentile, mean and SEM).

Figures 3, 4 illustrate the localization of the graph connector hubs at the alpha 2 and alpha 3 bands in the Nold and ADD groups. For sake of concision, such a localization was computed considering the 0.1 and 0.2 thresholds together. There was a certain spread of connector hubs over the scalp at those bands. However, LLC and iCoh measures showed convergent evidence of parietal connector hubs at alpha 2 and alpha 3 bands in both the Nold and ADD groups. Even using the most conservative criterion for the connector hub qualification (i.e., mean + 1 SD), consistent connector hubs for alpha 2 and alpha 3 bands were observed at parietal electrodes (i.e., P3, Pz, and P4). Notably, the iCoh values showed ***no substantial between-group differences in the topology of the parietal connector hubs at the alpha 2 and alpha 3 bands***.

### 3.5. Directionality of hubs from LLC and iCoh values at alpha 2 and alpha 3 bands

**Figure 5:**
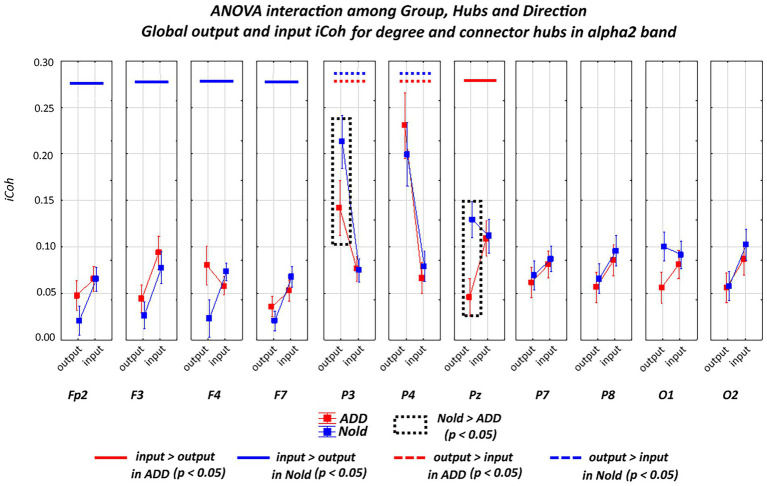
Global isolated lagged effective coherence (iCoh) values (mean across subjects ± SEM) in the alpha 2 frequency band within electrodes classified as substantial degree or connector hubs relative to a statistically significant ANOVA interaction (F [10, 750] = 2.42, *p* < 0.05) among the group (Nold and ADD), hubs (Fp2, F3, F4, F7, P3, P4, Pz, P7, P8, O1, and O2), and direction (output and input) factors. No statistically significant outliers were found according to Grubbs’ test (*p* < 0.0001).

**Figure 6:**
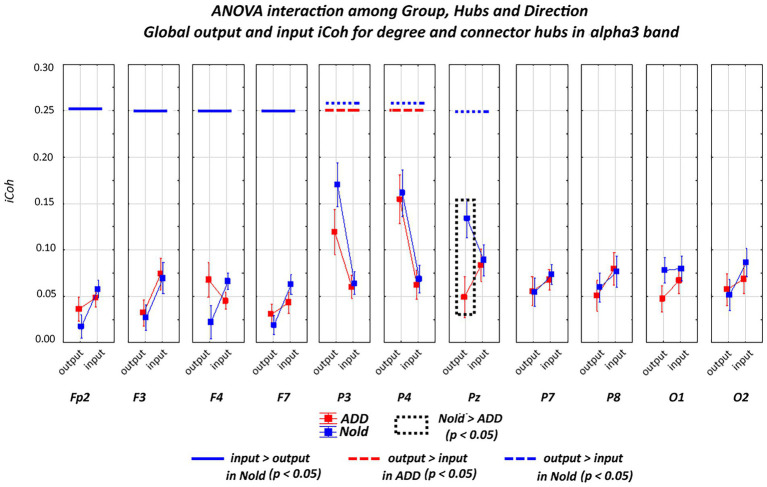
Global isolated lagged effective coherence (iCoh) values (mean across subjects ± SEM) in the alpha 3 frequency band within electrodes classified as substantial degree or connector hubs relative to a statistically significant ANOVA interaction (F [10, 750] = 2.48, *p* < 0.05) among the group (Nold and ADD), hubs (Fp2, F3, F4, F7, P3, P4, Pz, P7, P8, O1, and O2), and direction (output and input) factors. No statistically significant outliers were found according to Grubbs’ test (*p* < 0.0001).

Figures 5, 6 plot the global output (outward) and input (inward) iCoh alpha 2 and alpha 3 values at all electrodes denoted as a degree or a connector hub in the above analysis. Results showed a statistically significant ANOVA interaction of the iCoh alpha 2 values among group (Nold and ADD; independent variable), hub (electrodes with hub features), and direction (output and input) factors (*F*[10, 750] = 2.42, *p* < 0.05). Compared with the Nold group, the ADD group showed lower output global iCoh alpha 2 values at parietal electrodes (i.e., P3 and Pz; *p* < 0.05).

A statistically significant ANOVA interaction of the iCoh alpha 3 values was also observed among group, hub, and direction (*F*[10, 750] = 2.48, *p* < 0.05). Compared with the Nold group, the ADD group showed lower output global iCoh alpha 3 values at one parietal electrode (i.e., Pz; *p* < 0.05).

Additionally, a statistically significant ANOVA interaction of the iCoh alpha 3 values was observed among group, hub, and direction (*F* = 2.48, *p* < 0.05). Compared with the Nold group, the ADD group showed lower output global iCoh alpha 3 values at one parietal electrode (i.e., Pz; *p* < 0.05).

Supplementary material Table 1 reports the results of the Duncan planned *post-hoc* (*p* < 0.05) test relative to the ANOVA interaction effects. Of note, the above results were not caused by outliers, as shown by Grubbs’ test with an arbitrary threshold of *p* > 0.001 (see Supplementary Figure 10).

Overall, the above iCoh results showed ***no substantial between-group differences in the topology of the iCoh alpha 2 and alpha 3 values, with those values being maximized at parietal electrodes. However, the output iCoh alpha 2 and alpha 3 values at parietal electrodes were lower in the ADD group than in the Nold group***.

### 3.6. Control analysis on parietal connector hubs identified at alpha 2 and alpha 3 bands by other graph theory measures

To control for the robustness of the present results about the parietal connector hubs computed at the alpha 2 and alpha 3 bands, we used the following additional graph measures and definitions of those hubs (GraphVar 2.0 platform). According to Cole et al. (2015), a connector hub can be associated with high values of ND, PC, and Betweenness Centrality (BC). Notably, BC of a node is defined as the number of shortest graph paths that goes through that node (Rubinov and Sporns, 2010). According to Power et al. (2013), a connector hub can be associated with high values of the within-module degree z-score and PC.

Figures 3, 4 illustrate the results of this control analysis. There was again a certain spread of connector hubs over the scalp at alpha 2 and alpha 3 bands. However, convergent results showed ***significant connector hubs located at the parietal electrodes (i.e., P3, Pz, and P4) in both the Nold and ADD groups***. Supplementary material Tables 14–25 report detailed results of this control analysis.

### 3.7. Control analysis of the influence of normalized rsEEG spectral power density on iCoh values

To evaluate the influence of potential intergroup differences in the rsEEG spectral power density on iCoh (and LLC as the second interdependency measure) values, we included a control analysis for the comparison of the rsEEG power spectra for each frequency band of interest between the Nold and ADD groups. To this aim, we used the normalized rsEEG spectral power density calculated at each individual frequency band of interest (from delta to gamma) as the dependent variable in an ANOVA design, with group (Nold and ADD; independent variable), ROI (frontal, central, parietal, temporal, and occipital), and band (delta, theta, alpha 1, alpha 2, alpha 3, beta 1, beta 2, and gamma) as factors. The ROI were defined as those used for the interhemispheric iCoh analysis. We used the Log10-transformed rsEEG spectral power density values to meet the requirement of Gaussian distribution of the dependent ANOVA variable.

Results are illustrated in Supplementary Figure 11. Compared with the Nold group, the ADD group was characterized by (1) lower widespread alpha 2 and alpha 3 spectral power density values, especially at parietal and occipital ROI, and (2) higher widespread delta spectral power density values (*p* < 0.05).

Owing to the above-mentioned intergroup differences, we repeated the main statistical analyses by introducing the global alpha 2 or the alpha 3 regional normalized spectral power density as a covariate. Global values were calculated by averaging the regional values (as intergroup differences in the alpha 2 and alpha 3 bands were widespread). Results confirmed the previous main findings except for the intrahemispheric (non-directional) LLC values (no statistically significant interaction among the factors group, ROI, and band with global alpha 2 or alpha 3 spectral power densities as covariates; *p* > 0.05). In detail, the following results were obtained:

- intrahemispheric (non-directional) mean iCoh—covariate, global alpha 2 spectral power density: a statistically significant ANOVA interaction (*F*[28, 2072] = 3.55, *p* < 0.05) among the factors group, hemisphere, ROI, and band. Again, no significant *post-hoc* effect was observed in the planned tests (*p* > 0.05).
- intrahemispheric (non-directional) mean iCoh—covariate, global alpha 3 spectral power density: a statistically significant ANOVA interaction (*F*[28, 2072] = 3.02, *p* < 0.05) among the factors group, hemisphere, ROI, and band. Again, no significant *post-hoc* effect was observed in the planned tests (*p* > 0.05).
- intrahemispheric (non-directional) absolute difference iCoh—covariate, global alpha 2 spectral power density: a statistically significant ANOVA interaction (*F*[28, 2072] = 2.81, *p* < 0.05) among the factors group, hemisphere, ROI, and band. Again, no significant *post-hoc* effect was observed in the planned tests (*p* > 0.05).
- intrahemispheric (non-directional) absolute difference iCoh—covariate, global alpha 3 spectral power density: a statistically significant ANOVA interaction (*F*[28, 2072] = 2.02, *p* < 0.05) among the factors group, hemisphere, ROI, and band. Again, no significant *post-hoc* effect was observed in the planned tests (*p* > 0.05).
- interhemispheric LLC—covariate, global alpha 2 spectral power density: a statistically significant ANOVA interaction (*F*[28, 2072] = 2.22, *p* < 0.05) among the factors group, ROI, and band. A planned Duncan *post-hoc* test showed that, compared with the Nold group, the ADD group was mainly characterized by (1) lower LLC alpha 2 and alpha 3 values at the parietal and temporal ROI and (2) higher LLC delta values at frontal, parietal, and occipital ROI (*p* < 0.05).
- interhemispheric LLC—covariate, global alpha 3 spectral power density: a statistically significant ANOVA interaction (*F*[28, 2072] = 2.19, *p* < 0.05) among the factors group, ROI, and band. A planned Duncan *post-hoc* test showed that, compared with the Nold group, the ADD group was mainly characterized by (1) lower LLC alpha 2 and alpha 3 values at the parietal and temporal ROI and (2) higher LLC delta values at frontal, parietal, and occipital ROI (*p* < 0.05).

With regard to the directionality of hubs from iCoh values at alpha 2 and alpha 3 bands, we used the global alpha 2 and alpha 3 spectral power densities, respectively, as covariates in the main statistical analysis. Results with the global alpha 2 spectral power density as covariate confirmed a statistically significant ANOVA interaction of the iCoh alpha 2 values among group (Nold and ADD; independent variable), hub (electrodes with hub features), and direction (output and input) factors (*F*[10, 70] = 1.97, *p* < 0.05). Compared with the Nold group, the ADD group showed lower output global iCoh alpha 2 values at parietal electrodes (i.e., P3 and Pz; *p* < 0.05). No statistically significant ANOVA interaction (*p* > 0.05) was observed, including with global alpha 3 spectral power density as a covariate.

## 4. Discussion

### 4.1. Converging evidence of LLC and iCoh measures about the interdependencies of rsEEG rhythms at electrode pairs

In the present study, both bivariate LLC and multivariate iCoh measures showed that, compared with the Nold participants, the ADD patients were characterized by lower interdependencies of rsEEG ***alpha*** rhythms, especially at ***parietal*** electrode pairs. This effect was more spatially sharp with multivariate iCoh measures than with bivariate LLC measures. These results confirm the spatial variability of the effects derived from different techniques estimating interdependencies of rsEEG rhythms at electrode pairs and emphasize the importance of using more than one technique, including at least one multivariate approach (Blinowska, 2011; Blinowska et al., 2017).

The current results are globally in line with the bulk of previous rsEEG studies showing that ADD patients exhibit lower interrelatedness of rsEEG rhythms at alpha and higher frequencies at posterior electrode pairs (Leuchter et al., 1992, 1994; Besthorn et al., 1994; Dunkin et al., 1994; Sloan et al., 1994; Stam et al., 1995, 1996, 2003, 2009; Jelic et al., 1998, 2000; Locatelli et al., 1998; Anghinah et al., 2000; Knott et al., 2000; Adler et al., 2003; Babiloni et al., 2004a, 2006a, 2018; Pogarell et al., 2005; de Haan et al., 2009; Fonseca et al., 2011, 2013).

Additionally, the current results showed certain effects of ADD on the interdependencies of rsEEG ***delta*** rhythms at electrode pairs, but only when the ***bivariate*** LLC technique was used. Compared with the Nold group, the ADD group exhibited higher LLC values at frontal, parietal, and occipital delta rhythms. These effects were globally in agreement with previous rsEEG evidence obtained using bivariate techniques (e.g., FFT-based spectral coherence or LLC) to investigate the effects of ADD on the interdependencies of rsEEG rhythms (Locatelli et al., 1998; Babiloni et al., 2010, 2018; Hsiao et al., 2013, 2014). Given the lack of effects of ADD on interdependencies of rsEEG delta rhythms derived from multivariate iCoh measures, we did not estimate hubs at delta rhythms. Notably, the results at delta rhythms indicated that the present iCoh measures were not redundant compared with those of spectral power density, which showed greater widespread rsEEG delta power in the ADD group than the Nold group.

### 4.2. Converging evidence of LLC and iCoh measures about graph hubs at parietal electrodes and alpha rhythms

The present alpha LLC and iCoh measures showed converging evidence of prominent ***degree and connector hubs*** at ***parietal*** electrode pairs (i.e., P3, Pz, and P4). Furthermore, both groups were characterized by a ***prominent*** alpha iCoh ***outward direction*** (output or outflow) from parietal electrodes to other electrodes exhibiting degree hub properties. This effect was lower in the ADD group than in the Nold group and was consistent with the three definitions of connector hubs (Rubinov and Sporns, 2010; Power et al., 2013; Cole et al., 2015).

Taken together, it can be speculated that the present alpha LLC-iCoh and graph results may reflect abnormalities in parietal networks underpinning the regulation of quiet vigilance in ADD patients but with a global preservation of the parietal hub function, as revealed by the present analysis of the rsEEG alpha rhythms.

In line with this speculation, previous studies investigating directional interdependencies of rsEEG rhythms in cognitively unimpaired adults showed prominent outflow measures at alpha (and beta) rhythms from parietal electrodes, based on the computation of multivariate directed transfer function (Kuś et al., 2004; Blinowska and Kaminski, 2013). These measures were reduced in relation to a decrease in vigilance and an increase in errors during a continuous cognitive task in those adults (Liu et al., 2010). When applied to AD patients, directed transfer function measures at rsEEG alpha (and beta) rhythms exhibited lower outflow from parietal to frontal electrodes in ADD and ADMCI patients than in Nold participants (Babiloni et al., 2008, 2009a,b; Blinowska et al., 2017). These effects might be partially caused by abnormal ascending inputs coming from the thalamic and cholinergic basal forebrain regions (Hughes and Crunelli, 2005; Babiloni et al., 2006a, 2009a,b, 2020a, 2021; Wan et al., 2019).

### 4.3. What might graph hubs from rsEEG alpha rhythms tell us about add patients?

At this early stage of the research, we can only speculate about the neurophysiological significance of the present results. As novel and original neurophysiological findings, the present study showed evidence of prominent parietal hubs from interdependencies of the rsEEG alpha rhythms but with reduced outward iCoh measures in AD patients with mild-to-moderate dementia (mean MMSE score of approximately 19/30). An exciting hypothesis for future longitudinal studies is that the AD progression to severe dementia might be associated with (1) outward alpha iCoh measures that are even more reduced from parietal electrodes, (2) loss of degree and connector hubs from the present LLC and iCoh measures, and (3) increased disorders in the regulation and maintenance of quiet vigilance during the daytime, with frequent episodes of drowsiness, misperceptions, and light sleep. If confirmed, the present rsEEG evidence of partially preserved parietal hubs in mild-to-moderate ADD patients would reflect a sort of resilience or initial vulnerability of the brain networks underpinning quiet vigilance.

In line with this speculation, previous rsEEG evidence showed several signs of topographically widespread impairment of brain networks in ADD patients, as revealed by graph theory indexes. Multivariate directional techniques based on a Granger causality matrix from rsEEG alpha and beta rhythms unveiled that ADD patients were globally characterized by lower global efficiency, increased local efficiency, and lower resilience of cortical networks (Afshari and Jalili, 2017). Furthermore, these patients were characterized by lower inward and outward directions of interdependencies of the whole-band rsEEG activity recorded from posterior electrodes, with maximum abnormalities of degree hubs at parietal electrodes (Franciotti et al., 2019), but not replicated with a bivariate mutual information technique (Franciotti et al., 2022).

In the ***resting state*** condition, prominent outward directionality of the present hubs at parietal electrodes and alpha rhythms might reflect the synchronization and interdependence of neural activity into posterior thalamocortical and corticothalamic loops, which might maintain cortical arousal underpinning vigilance against sleep intrusion (Hughes and Crunelli, 2005; Lörincz et al., 2008, 2009; Crunelli et al., 2015). Indeed, the greater the posterior rsEEG alpha rhythms, the greater the ***cortical inhibition*** in quiet vigilance, the lower the attention to external stimuli (Pfurtscheller and Klimesch, 1992; Boksem et al., 2005; Babiloni et al., 2020b). Those thalamic and cortical reciprocal interactions might influence several cortical areas, including the nodes of the ‘***default mode network***’ (Raichle et al., 2001; Buckner et al., 2008). Overall, such traveling alpha rhythms may flow from higher-to lower-order areas in the visual and somatosensory cortices (Halgren et al., 2019). The effect may be to facilitate the scanning of internal and external environments (Liu et al., 2010; Al-Shargie et al., 2019), extract relevant features on demand (Ermentrout and Kleinfeld, 2001), and support communications within nodes of brain networks in relation to vigilance (Han et al., 2008; Crunelli et al., 2018).

### 4.4. Methodological limitations of the present study

This study was not performed within a unique multicentric clinical trial, so the present recording units did not follow the identical clinical, neuropsychological, and neuroimaging procedures during the enrollment of Nold and ADD participants. This makes the present study exploratory in nature.

Standard biomarkers of AD neuropathology (e.g., cerebrospinal diagnostic measures of Ab42/phospho tau or amyloid positron emission tomography) were not systematically measured in the present Nold and ADD participants, so only the strongest and most robust results could emerge at the group level. This limitation may explain some significant variability of graph indexes at rsEEG delta rhythms.

We used a low number of scalp electrodes to record rsEEG activity (i.e., 19 electrodes placed according to 10–20 system), two standard bivariate (LLC) and multivariate (iCoh) techniques estimating the interrelatedness of the rsEEG activity at electrode pairs, and well-known graph indexes in line with the general methodology of several previous successful studies; those studies investigated the graph-based rsEEG topology in ADD patients based on ‘synchronization likelihood’, ‘phase lag index’, ‘synchronization likelihood’, ‘generalized composite multiscale entropy vector’, and ‘mutual information’ techniques applied to rsEEG data recorded from ≤19 scalp electrodes (Stam et al., 2007a; De Haan et al., 2009; Engels et al., 2015; Yu et al., 2016; Song et al., 2019; Das and Puthankattil, 2022; Franciotti et al., 2022).

This intrinsic low resolution of the present rsEEG approach was partially considered by averaging the LLC and iCoh measures in large scalp ROI. Furthermore, head volume conduction effects may inflate LLC and iCoh measures. Indeed, electric fields can instantaneously spread from a brain source to several scalp electrodes, thus generating spurious (fake) interdependencies of rsEEG rhythms at electrode pairs. These effects of head volume conduction are partially mitigated by the fact that LLC and iCoh measures are insensitive to zero-lag interdependencies of rsEEG rhythms. However, the present application of those techniques at scalp electrodes ignores observational equations modeling confounding effects of head volume conduction and position/orientation of cortical sources of scalp EEG activity (Babiloni et al., 2020). Therefore, confounding non-zero-lag head volume conduction effects and false ‘interrelatedness’ cannot be excluded in the interpretation of the present results. In this framework, it should be remarked that bivariate techniques (including LLC) may be more prone to those confounds than multivariate techniques (including iCoh), as the latter typically remove common correlations of the rsEEG activity among the electrode pairs (Blinowska and Kaminski, 2013; Babiloni et al., 2020b).

The intrinsic methodological limits of all bivariate and multivariate techniques (including LLC and iCoh) were recently discussed by an Expert Panel of the International Federation of Clinical Neurophysiology (IFCN; Babiloni et al., 2020b). The Expert Panel agreed that all bivariate (e.g., LLC, synchronization likelihood, phase lag index, etc.) and multivariate (e.g., iCoh, directed transfer function, etc.) techniques estimating the interrelatedness of the rsEEG activity at scalp electrode pairs may be subject to unmodeled effects of (1) brain neural populations ‘invisible’ to EEG recordings and (2) head volume conduction. Furthermore, the Expert Panel shared the following recommendations to fruitfully tackle (Babiloni et al., 2020b): (1) the use of the locution ‘*measures of the interrelatedness of rsEEG activity at scalp electrodes*’ rather than locutions such as “*measures of cortical functional connectivity from rsEEG activity*” to emphasize that the head volume conduction effects cannot be entirely taken into account when those techniques are applied at scalp electrode pairs; (2) the development of exploratory rsEEG studies carried out by investigators belonging to independent research institutions, to ensure a significant intersubjectivity in the interpretation of the results; (3) the use of at least two independent techniques for estimating the interrelatedness of the rsEEG activity at scalp electrodes, to compare the results and represent their intrinsic variability dependent on the methodology used; and (4) the exploitation of open science to cross-validate the research results using, when possible, freeware techniques validated by independent research groups. We grounded the present study design on these recommendations.

Keeping in mind the previously mentioned low spatial resolution and head volume conduction effects, we included a relatively low number of network nodes (corresponding to the standard 10–20 electrode montage) in the graph analysis. This low-resolution EEG method could not allow the disentanglement of the contribution of the nodes of the default mode network or associate parietal cortex. Therefore, future studies may improve the methodological approach with the following solutions: (1) large samples of the enrolled ADD, ADMCI, and Nold participants and a longitudinal design to enhance the statistical power of the study and test the impact of disease severity and progression on the topology of the interrelatedness of rsEEG activity; (2) harmonized protocols in the multicentric studies; (3) >48 scalp electrodes for the rsEEG recordings; (4) mathematical source and head volume conduction models for an rsEEG source estimation probing the activity of more cortical nodes, including those located in the default mode and other relevant cortical networks; (5) a multimodal approach, including the rs-fMRI recordings, to correlate the AD-related abnormal topology of the cortical functional connectivity, as revealed by rs-fMRI and rsEEG data; and (6) a more systematic variation of statistical thresholds to qualify the significant associations between sensors and the criteria used to define and describe the present hubs with those thresholds.

## 5. Conclusion

In the present exploratory study, we compared hubs modeled from measures of interdependencies of between-electrode rsEEG alpha rhythms in Nold and mild-to-moderate ADD participants. We tested the hypothesis of abnormal posterior hubs from those measures in ADD versus Nold participants. To report robust results, we measured interdependencies of rsEEG rhythms using both bivariate LLC and multivariate (directional) iCoh measures. Furthermore, we used three different definitions of ‘connector’ hub.

Convergent results of LLC and iCoh measures showed that in both Nold and ADD groups there were significant ‘degree’ and ‘connector’ hubs at ***parietal*** electrodes derived from rsEEG ***alpha rhythms***. Furthermore, these hubs showed a prominent outward directionality in both groups of participants. As a main difference between the two groups, the outward ‘directionality’ of the hubs at parietal electrodes was lower in the ADD group than in the Nold group.

Future longitudinal high-resolution rsEEG studies in ADD patients will have to test hypotheses about the resilience or vulnerability of those parietal hubs derived from rsEEG alpha rhythms and their relationships with the neuropathological burden, derangement in the DMN, and the neurophysiological regulation and maintenance of quiet vigilance during daytime.

## Data availability statement

The datasets presented in this article are not readily available because they may be made available by CB through a formal data sharing agreement and approval from the requesting researcher’s local ethics committee. The presentation of a formal collaboration project outline is also suggested. Requests to access the datasets should be directed to SL, susanna.lopez@uniroma1.it.

## Ethics statement

The studies involving human participants were reviewed and approved by Sapienza University of Rome. The patients/participants provided their written informed consent to participate in this study.

## Author contributions

CBa and SL: conceptualization, methodology, formal analysis, investigation, data curation, validation, project administration, writing—original draft, supervision, writing—review and editing. CP, GN, and RL: formal analysis, software, writing—review and editing. AP, FN, DA, FF, DM, AC, GK, AB, MO, BB, AS, RF, CBu, FG, BG, GY, FS, LV, and LB: methodology, project administration, writing—review and editing. All authors contributed to the article and approved the submitted version.

## Funding

In this study, the electroencephalographic data analysis was partially supported by the funds of “Ricerca Corrente 2021–2022” attributed by the Ministry of Health, Italy to the IRCCS Synlab SDN of Naples, IRCCS OASI Maria SS of Troina, IRCCS San Giovanni di Dio “Fatebenefratelli” of Brescia, and IRCCS San Raffaele Rome.

## Conflict of interest

The authors declare that the research was conducted in the absence of any commercial or financial relationships that could be construed as a potential conflict of interest.

## Publisher’s note

All claims expressed in this article are solely those of the authors and do not necessarily represent those of their affiliated organizations, or those of the publisher, the editors and the reviewers. Any product that may be evaluated in this article, or claim that may be made by its manufacturer, is not guaranteed or endorsed by the publisher.

## Footnotes

^1^ https://www.uzh.ch/keyinst/loreta

^2^ https://www.nitrc.org/projects/graphvar

^3^ http://adni.loni.usc.edu

^4^ www.statsoft.com
