# Supplementary figures and images for: Patients with Alzheimer’s disease dementia show partially preserved parietal ‘hubs’ modeled from resting-state alpha electroencephalographic rhythms
Source: Front Aging Neurosci. 2023 Jan 26;15:780014. doi: 10.3389/fnagi.2023.780014 (PMC9908964; doi:10.3389/fnagi.2023.780014)

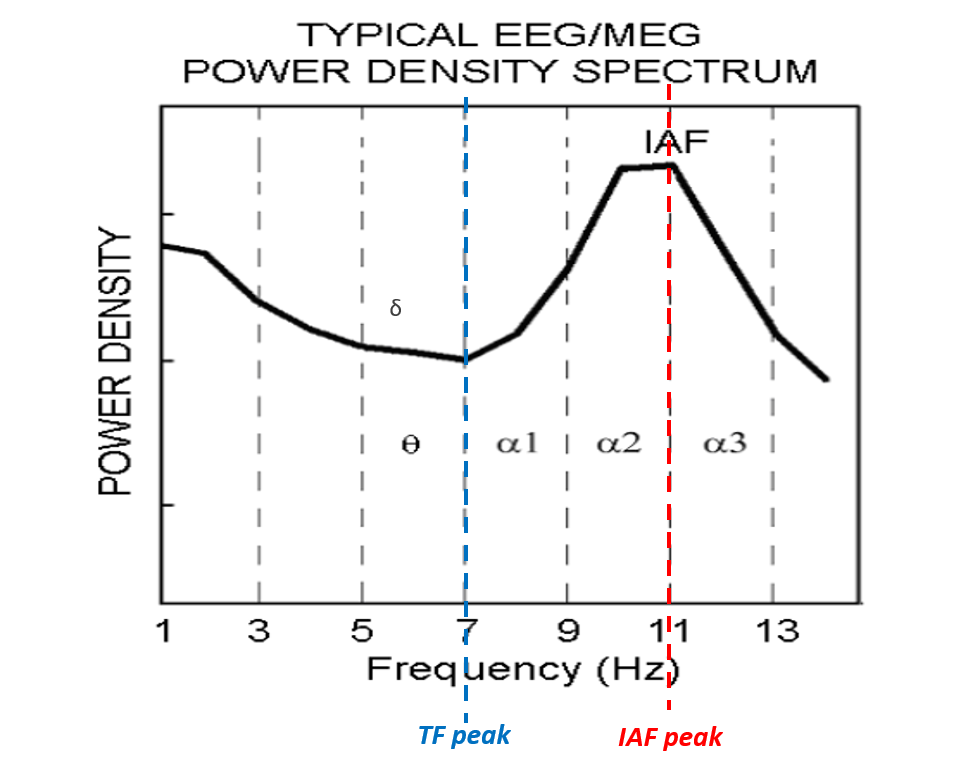

Supplement: Supplementary file 1 [file Image_1.TIF]

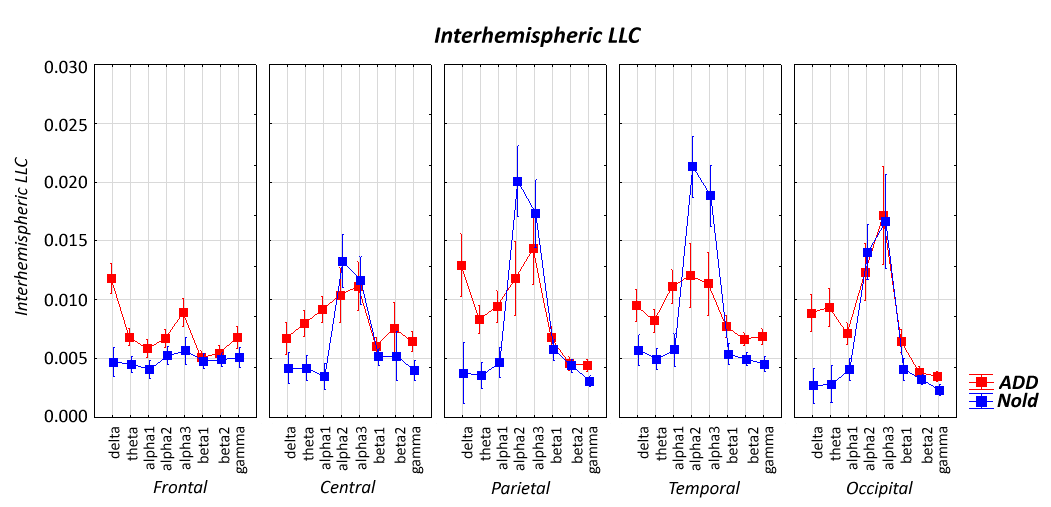

Supplement: Supplementary file 2 [file Image_2.TIF]

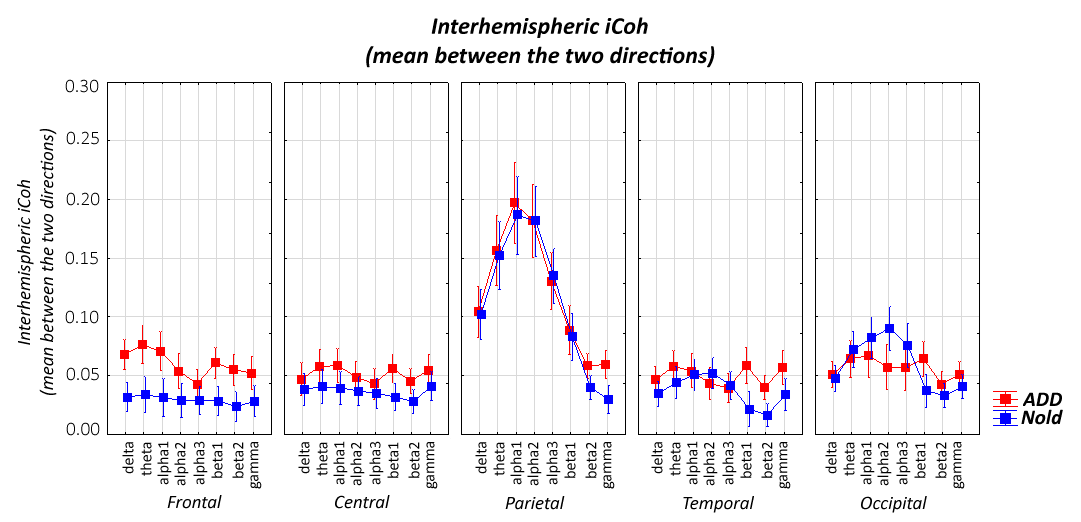

Supplement: Supplementary file 3 [file Image_3.TIF]

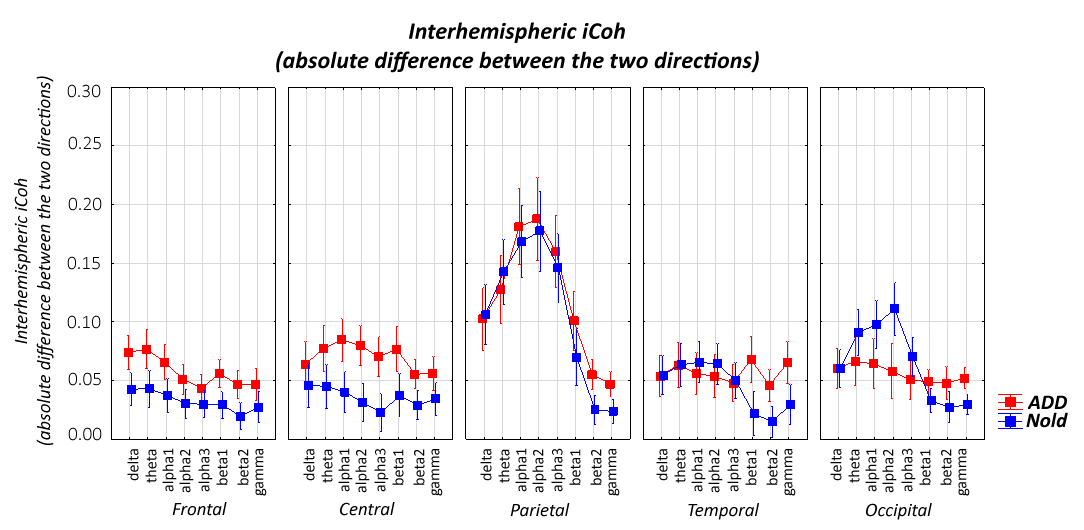

Supplement: Supplementary file 4 [file Image_4.TIF]

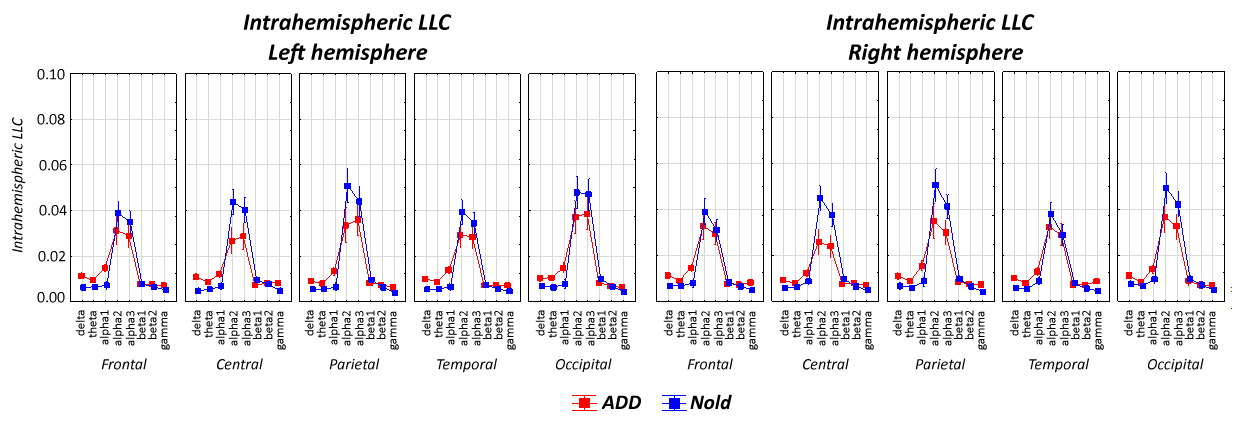

Supplement: Supplementary file 5 [file Image_5.TIF]

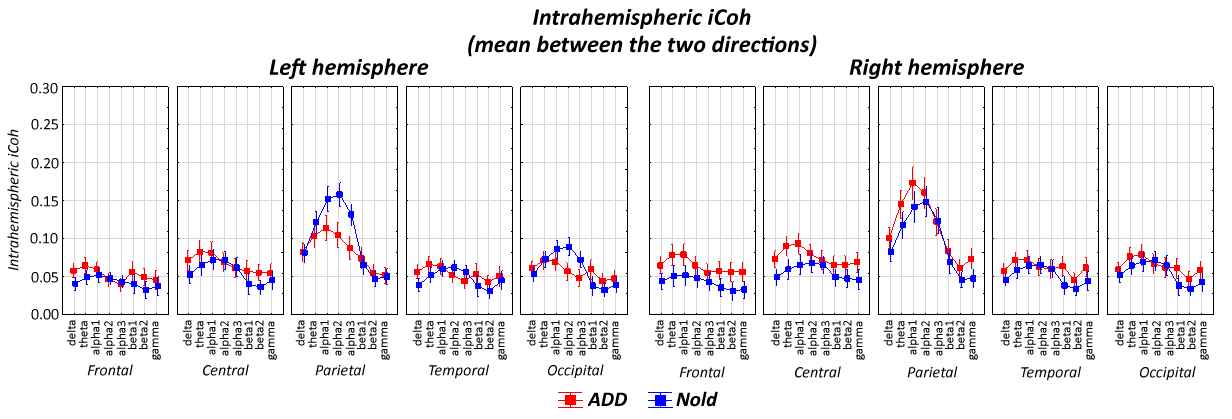

Supplement: Supplementary file 6 [file Image_6.TIF]

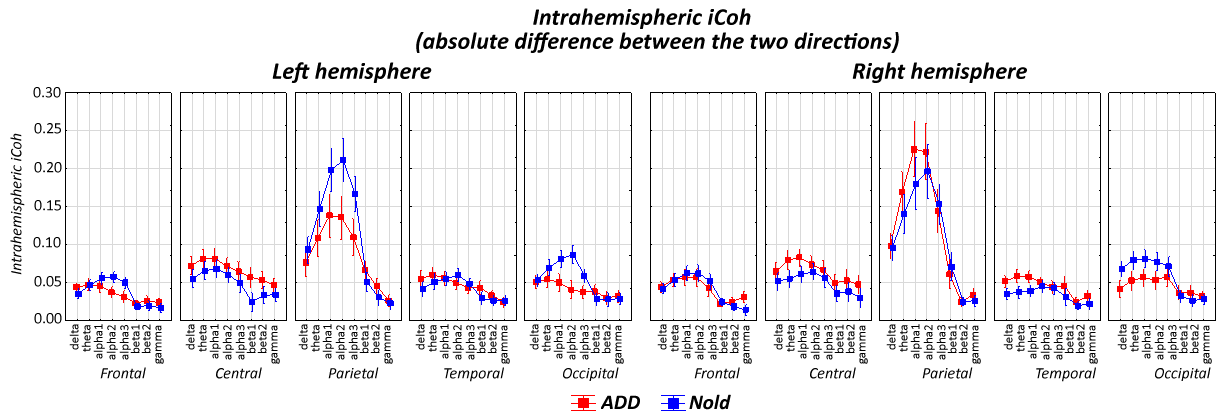

Supplement: Supplementary file 7 [file Image_7.TIF]

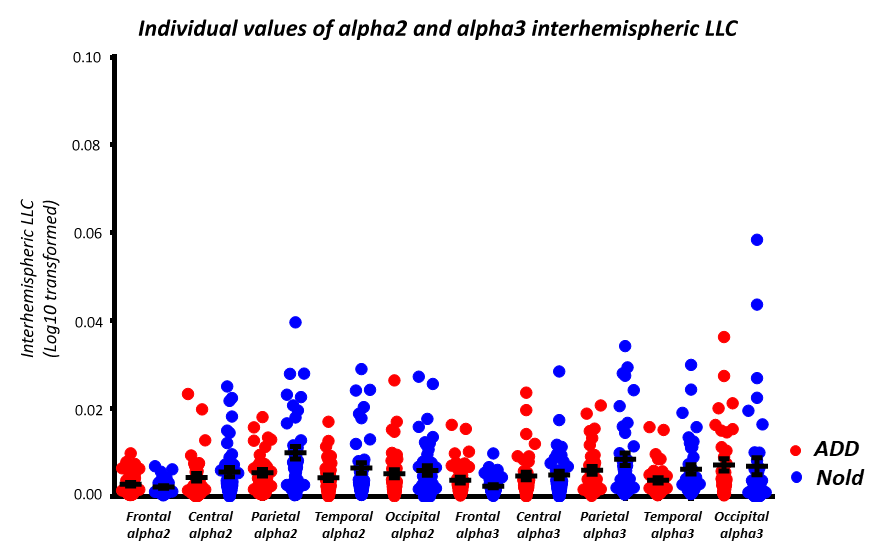

Supplement: Supplementary file 8 [file Image_8.TIF]

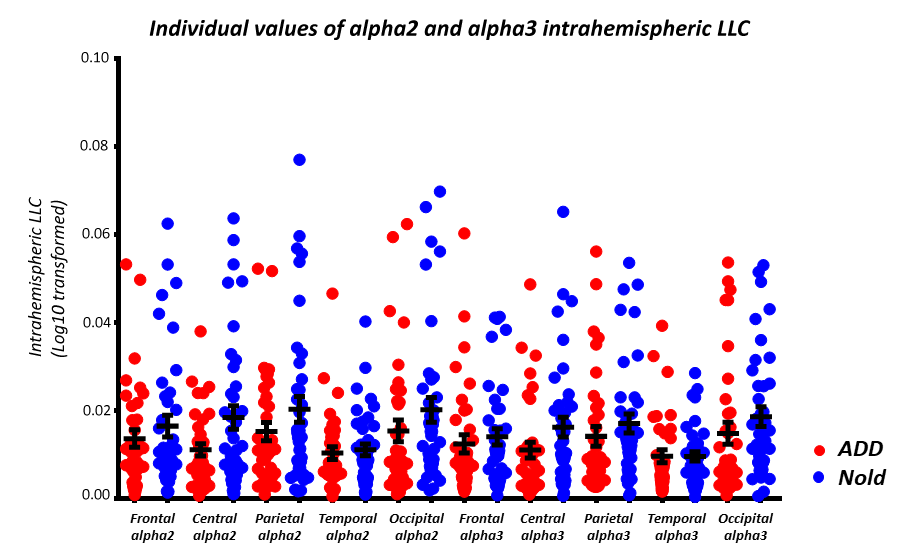

Supplement: Supplementary file 9 [file Image_9.TIF]

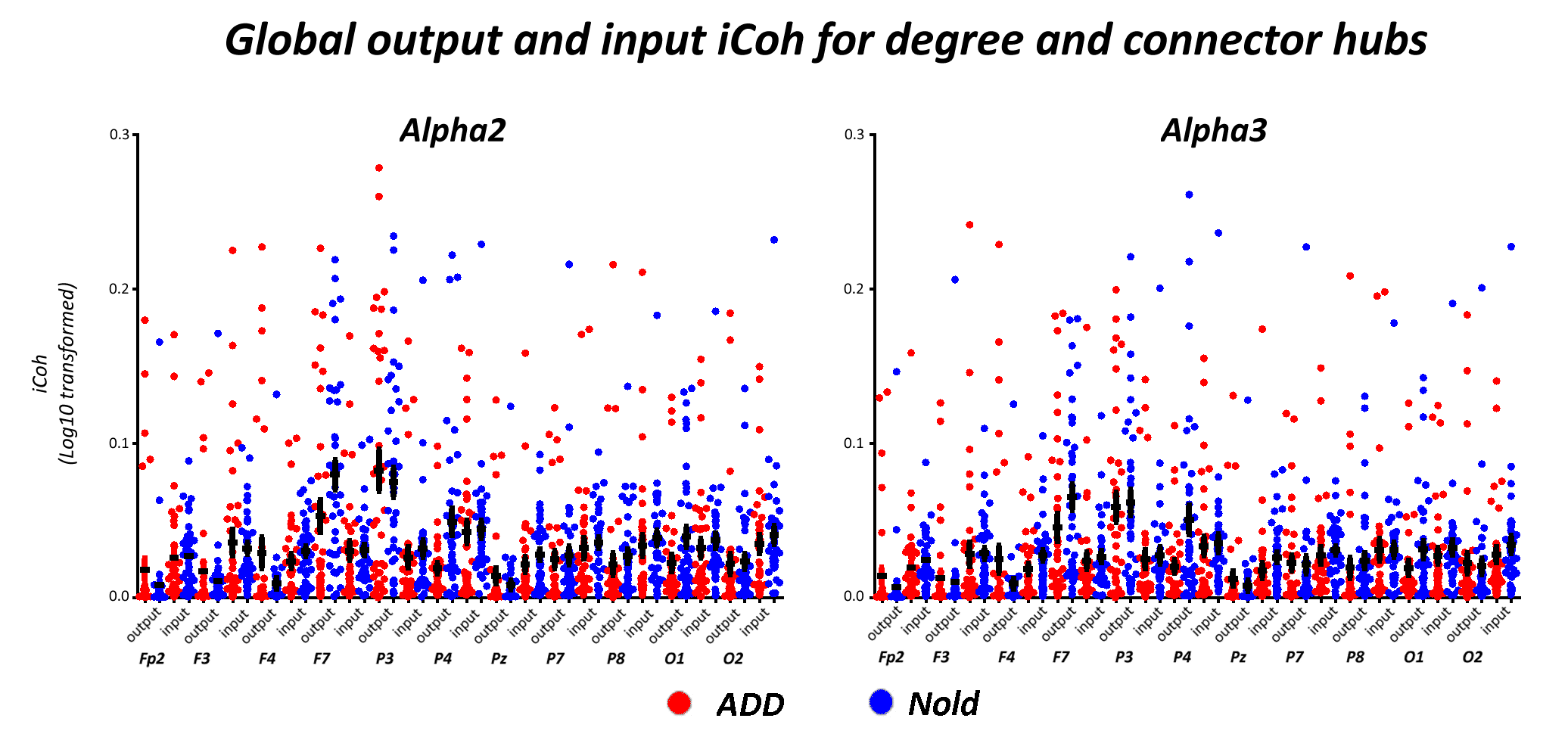

Supplement: Supplementary file 10 [file Image_10.TIF]

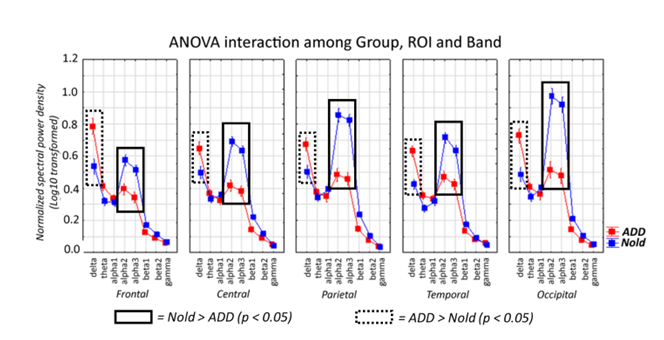

Supplement: Supplementary file 11 [file Image_11.TIF]
