# Supplementary material for: Patients with Alzheimer’s disease dementia show partially preserved parietal ‘hubs’ modeled from resting-state alpha electroencephalographic rhythms
Source: Front Aging Neurosci. 2023 Jan 26;15:780014. doi: 10.3389/fnagi.2023.780014 (PMC9908964; doi:10.3389/fnagi.2023.780014)
Supplement: Supplementary file 12 [file Data_Sheet_1.docx]

**Supplementary Materials**

**Supplementary Materials Table legends**

**Table SM1.** Post-hoc p-values (Duncan test) relative to the ANOVA interaction effects on the interhemispheric and intrahemispheric Linear Lagged Connectivity (LLC).

**Table SM2.** Degree hubs for the Linear Lagged Connectivity (LLC) values in the Nold and ADD groups for the alpha2 band, as defined by the ND with the different criteria (mean + 1SD, 80^th^ percentile, 70^th^ percentile, mean + 1SEM).

**Table SM3.** Degree hubs for the Linear Lagged Connectivity (LLC) values in the Nold and ADD groups for the alpha3 band, as defined by the ND with the different criteria (mean + 1SD, 80^th^ percentile, 70^th^ percentile, mean + 1SEM).

**Table SM4.** Degree hubs for the mean isolated lagged effective Coherence (iCoh) values in the Nold and ADD groups for the alpha2 band, as defined by the ND with the different criteria (mean + 1SD, 80^th^ percentile, 70^th^ percentile, mean + 1SEM).

**Table SM5.** Degree hubs for the mean isolated lagged effective Coherence (iCoh) values in the Nold and ADD groups for the alpha3 band, as defined by the ND with the different criteria (mean + 1SD, 80^th^ percentile, 70^th^ percentile, mean + 1SEM).

**Table SM6.** Degree hubs for the absolute difference isolated lagged effective Coherence (iCoh)values in the Nold and ADD groups for the alpha2 band, as defined by the ND with the different criteria (mean + 1SD, 80^th^ percentile, 70^th^ percentile, mean + 1SEM).

**Table SM7.** Degree hubs for the absolute difference isolated lagged effective Coherence (iCoh)values in the Nold and ADD groups for the alpha3 band, as defined by the ND with the different criteria (mean + 1SD, 80^th^ percentile, 70^th^ percentile, mean + 1SEM).

**Table SM8.** Connector and provincial hubs for the alpha2 Linear Lagged Connectivity (LLC) values in the Nold and ADD groups. Hubs were defined according to Nodal Degree (ND) and classified in connector and provincial hubs defined by the Participation Coefficient (PC) and Clustering Coefficient (CC) calculated at 0.1 and 0.2 graph thresholds. Specifically, connector hubs were defined by a high PC and low CC, while provincial hubs were defined by a low PC and high CC, with different criteria (mean +/- 1SD, 80^th^/20^th^ percentile, 70^th^/30^th^ percentile, mean +/- 1SEM). Percentiles corresponding to Standard Deviation (SD) and Standard Error Mean (SEM) values in a normal distribution are reported for sake of clarity.

**Table SM9.** Connector and provincial hubs for the alpha3 Linear Lagged Connectivity (LLC) values in the Nold and ADD groups. Hubs were defined according to Nodal Degree (ND) and classified in connector and provincial hubs defined by the Participation Coefficient (PC) and Clustering Coefficient (CC) calculated at 0.1 and 0.2 graph thresholds. Specifically, connector hubs were defined by a high PC and low CC, while provincial hubs were defined by a low PC and high CC, with different criteria (mean +/- 1SD, 80^th^/20^th^ percentile, 70^th^/30^th^ percentile, mean +/- 1SEM). Percentiles corresponding to Standard Deviation (SD) and Standard Error Mean (SEM) values in a normal distribution are reported for sake of clarity.

**Table SM10.** Connector and provincial hubs for the alpha2 mean isolated lagged effective Coherence (iCoh) values in the Nold and ADD groups. Hubs were defined according to Nodal Degree (ND) and classified in connector and provincial hubs defined by the Participation Coefficient (PC) and Clustering Coefficient (CC) calculated at 0.1 and 0.2 graph thresholds. Specifically, connector hubs were defined by a high PC and low CC, while provincial hubs were defined by a low PC and high CC, with different criteria (mean +/- 1SD, 80^th^/20^th^ percentile, 70^th^/30^th^ percentile, mean +/- 1SEM). Percentiles corresponding to Standard Deviation (SD) and Standard Error Mean (SEM) values in a normal distribution are reported for sake of clarity.

**Table SM11.** Connector and provincial hubs for the alpha3 mean isolated lagged effective Coherence (iCoh) values in the Nold and ADD groups. Hubs were defined according to Nodal Degree (ND) and classified in connector and provincial hubs defined by the Participation Coefficient (PC) and Clustering Coefficient (CC) calculated at 0.1 and 0.2 graph thresholds. Specifically, connector hubs were defined by a high PC and low CC, while provincial hubs were defined by a low PC and high CC, with different criteria (mean +/- 1SD, 80^th^/20^th^ percentile, 70^th^/30^th^ percentile, mean +/- 1SEM). Percentiles corresponding to Standard Deviation (SD) and Standard Error Mean (SEM) values in a normal distribution are reported for sake of clarity.

**Table SM12.** Connector and provincial hubs for the alpha2 absolute difference isolated lagged effective Coherence (iCoh) values in the Nold and ADD groups. Hubs were defined according to Nodal Degree (ND) and classified in connector and provincial hubs defined by the Participation Coefficient (PC) and Clustering Coefficient (CC) calculated at 0.1 and 0.2 graph thresholds. Specifically, connector hubs were defined by a high PC and low CC, while provincial hubs were defined by a low PC and high CC, with different criteria (mean +/- 1SD, 80^th^/20^th^ percentile, 70^th^/30^th^ percentile, mean +/- 1SEM). Percentiles corresponding to Standard Deviation (SD) and Standard Error Mean (SEM) values in a normal distribution are reported for sake of clarity.

**Table SM13.** Connector and provincial hubs for the alpha3 absolute difference isolated lagged effective Coherence (iCoh) values in the Nold and ADD groups. Hubs were defined according to Nodal Degree (ND) and classified in connector and provincial hubs defined by the Participation Coefficient (PC) and Clustering Coefficient (CC) calculated at 0.1 and 0.2 graph thresholds. Specifically, connector hubs were defined by a high PC and low CC, while provincial hubs were defined by a low PC and high CC, with different criteria (mean +/- 1SD, 80^th^/20^th^ percentile, 70^th^/30^th^ percentile, mean +/- 1SEM). Percentiles corresponding to Standard Deviation (SD) and Standard Error Mean (SEM) values in a normal distribution are reported for sake of clarity.

**Table SM14**. Connector and provincial hubs for the alpha2 Linear Lagged Connectivity (LLC) values in the Nold and ADD groups. Hubs were defined according to Nodal degree and classified in connector and provincial hubs according to the Participation Coefficient (PC) and Betweenness Centrality (BC) calculated at 0.1 and 0.2 thresholds. Specifically, connector hubs were defined by a high PC and BC, while provincial hubs were defined by a low PC and BC, with different criteria (mean and SD, 80^th^/20^th^ percentile, 70^th^/30^th^ percentile, mean and SEM). Percentiles corresponding to Standard Deviation (SD) and Standard Error Mean (SEM) values in a normal distribution are reported for sake of clarity.

**Table SM15**. Connector and provincial hubs for the alpha3 Linear Lagged Connectivity (LLC) values in the Nold and ADD groups. Hubs were defined according to Nodal degree and classified in connector and provincial hubs according to the Participation Coefficient (PC) and Betweenness Centrality (BC) calculated at 0.1 and 0.2 thresholds. Specifically, connector hubs were defined by a high PC and BC, while provincial hubs were defined by a low PC and BC, with different criteria (mean and SD, 80^th^/20^th^ percentile, 70^th^/30^th^ percentile, mean and SEM). Percentiles corresponding to Standard Deviation (SD) and Standard Error Mean (SEM) values in a normal distribution are reported for sake of clarity.

**Table SM16**. Connector and provincial hubs for the alpha2 mean isolated lagged effective Coherence (iCoh) values in the Nold and ADD groups. Hubs were defined according to Nodal degree and classified in connector and provincial hubs according to the Participation Coefficient (PC) and Betweenness Centrality (BC) calculated at 0.1 and 0.2 thresholds. Specifically, connector hubs were defined by a high PC and BC, while provincial hubs were defined by a low PC and BC, with different criteria (mean and SD, 80^th^/20^th^ percentile, 70^th^/30^th^ percentile, mean and SEM). Percentiles corresponding to Standard Deviation (SD) and Standard Error Mean (SEM) values in a normal distribution are reported for sake of clarity.

**Table SM17**. Connector and provincial hubs for the alpha3 mean isolated lagged effective Coherence (iCoh) values in the Nold and ADD groups. Hubs were defined according to Nodal degree and classified in connector and provincial hubs according to the Participation Coefficient (PC) and Betweenness Centrality (BC) calculated at 0.1 and 0.2 thresholds. Specifically, connector hubs were defined by a high PC and BC, while provincial hubs were defined by a low PC and BC, with different criteria (mean and SD, 80^th^/20^th^ percentile, 70^th^/30^th^ percentile, mean and SEM). Percentiles corresponding to Standard Deviation (SD) and Standard Error Mean (SEM) values in a normal distribution are reported for sake of clarity.

**Table SM18**. Connector and provincial hubs for the alpha2 absolute difference isolated lagged effective Coherence (iCoh) values in the Nold and ADD groups. Hubs were defined according to Nodal degree and classified in connector and provincial hubs according to the Participation Coefficient (PC) and Betweenness Centrality (BC) calculated at 0.1 and 0.2 thresholds. Specifically, connector hubs were defined by a high PC and BC, while provincial hubs were defined by a low PC and BC, with different criteria (mean and SD, 80^th^/20^th^ percentile, 70^th^/30^th^ percentile, mean and SEM). Percentiles corresponding to Standard Deviation (SD) and Standard Error Mean (SEM) values in a normal distribution are reported for sake of clarity.

**Table SM19.** Connector and provincial hubs for the alpha3 absolute difference isolated lagged effective Coherence (iCoh) values in the Nold and ADD groups. Hubs were defined according to Nodal degree and classified in connector and provincial hubs according to the Participation Coefficient (PC) and Betweenness Centrality (BC) calculated at 0.1 and 0.2 thresholds. Specifically, connector hubs were defined by a high PC and BC, while provincial hubs were defined by a low PC and BC, with different criteria (mean and SD, 80^th^/20^th^ percentile, 70^th^/30^th^ percentile, mean and SEM). Percentiles corresponding to Standard Deviation (SD) and Standard Error Mean (SEM) values in a normal distribution are reported for sake of clarity.

**Table SM20**. Connector and provincial hubs for the alpha2 Linear Lagged Connectivity (LLC) values in the Nold and ADD groups. Connector and provincial hubs were defined by Within-module degree z-score and Participation Coefficient (PC) calculated at 0.1 and 0.2 thresholds. Specifically, connector hubs were defined by a high PC, while provincial hubs were defined by a low PC, with different criteria (mean and SD, 80^th^/20^th^ percentile, 70^th^/30^th^ percentile, mean and SEM). Percentiles corresponding to Standard Deviation (SD) and Standard Error Mean (SEM) values in a normal distribution are reported for sake of clarity.

**Table SM21**. Connector and provincial hubs for the alpha3 Linear Lagged Connectivity (LLC) values in the Nold and ADD groups. Connector and provincial hubs were defined by Within-module degree z-score and Participation Coefficient (PC) calculated at 0.1 and 0.2 thresholds. Specifically, connector hubs were defined by a high PC, while provincial hubs were defined by a low PC, with different criteria (mean and SD, 80^th^/20^th^ percentile, 70^th^/30^th^ percentile, mean and SEM). Percentiles corresponding to Standard Deviation (SD) and Standard Error Mean (SEM) values in a normal distribution are reported for sake of clarity.

**Table SM22**. Connector and provincial hubs for the alpha2 mean isolated lagged effective Coherence (iCoh) values in the Nold and ADD groups. Connector and provincial hubs were defined by Within-module degree z-score and Participation Coefficient (PC) calculated at 0.1 and 0.2 thresholds. Specifically, connector hubs were defined by a high PC, while provincial hubs were defined by a low PC, with different criteria (mean and SD, 80^th^/20^th^ percentile, 70^th^/30^th^ percentile, mean and SEM). Percentiles corresponding to Standard Deviation (SD) and Standard Error Mean (SEM) values in a normal distribution are reported for sake of clarity.

**Table SM23**. Connector and provincial hubs for the alpha3 mean isolated lagged effective Coherence (iCoh) values in the Nold and ADD groups. Connector and provincial hubs were defined by Within-module degree z-score and Participation Coefficient (PC) calculated at 0.1 and 0.2 thresholds. Specifically, connector hubs were defined by a high PC, while provincial hubs were defined by a low PC, with different criteria (mean and SD, 80^th^/20^th^ percentile, 70^th^/30^th^ percentile, mean and SEM). Percentiles corresponding to Standard Deviation (SD) and Standard Error Mean (SEM) values in a normal distribution are reported for sake of clarity.

**Table SM24**. Connector and provincial hubs for the alpha2 absolute difference isolated lagged effective Coherence (iCoh) values in the Nold and ADD groups. Connector and provincial hubs were defined by Within-module degree z-score and Participation Coefficient (PC) calculated at 0.1 and 0.2 thresholds. Specifically, connector hubs were defined by a high PC, while provincial hubs were defined by a low PC, with different criteria (mean and SD, 80^th^/20^th^ percentile, 70^th^/30^th^ percentile, mean and SEM). Percentiles corresponding to Standard Deviation (SD) and Standard Error Mean (SEM) values in a normal distribution are reported for sake of clarity.

**Table SM25.** Connector and provincial hubs for the alpha3 absolute difference isolated lagged effective Coherence (iCoh) values in the Nold and ADD groups. Connector and provincial hubs were defined by Within-module degree z-score and Participation Coefficient (PC) calculated at 0.1 and 0.2 thresholds. Specifically, connector hubs were defined by a high PC, while provincial hubs were defined by a low PC, with different criteria (mean and SD, 80^th^/20^th^ percentile, 70^th^/30^th^ percentile, mean and SEM). Percentiles corresponding to Standard Deviation (SD) and Standard Error Mean (SEM) values in a normal distribution are reported for sake of clarity.

**Supplementary Materials Figure Legends**

**Figure SM1.** Definition of the individual frequency bands according to the Transition Frequency (TF) and Individual Alpha Frequency (IAF) hallmarks.

**Figure SM2**. Mean values (± Standard Error Mean, SEM) of the interhemisphericLinear Lagged Connectivity (LLC) values computed in relation to a statistical ANOVA interaction (F = 2.25, p < 0.05) among the factors Group (Nold, ADD), ROI (frontal, central, parietal, temporal and occipital) and Band (delta, theta, alpha 1, alpha 2, alpha 3, beta 1, beta 2, and gamma).

**Figure SM3**. Mean values (± Standard Error Mean, SEM) of the interhemispheric mean isolated lagged effective Coherence (iCoh) values. No statistically significant ANOVA interaction (p > 0.05) among the factors Group (Nold, ADD), ROI (frontal, central, parietal, temporal and occipital) and Band (delta, theta, alpha 1, alpha 2, alpha 3, beta 1, beta 2, and gamma) was observed.

**Figure SM4**. Mean values (± Standard Error Mean, SEM) of the interhemispheric absolute difference isolated lagged effective Coherence (iCoh) values. No statistically significant ANOVA interaction (p > 0.05) among the factors Group (Nold, ADD), ROI (frontal, central, parietal, temporal and occipital) and Band (delta, theta, alpha 1, alpha 2, alpha 3, beta 1, beta 2, and gamma) was observed.

**Figure SM5**. Mean values (± SEM) of the intrahemisphericLinear Lagged Connectivity (LLC) values computed in relation to a statistical ANOVA interaction (F = 3.32, p < 0.05) among the factors Group (Nold, ADD), ROI (frontal, central, parietal, temporal and occipital) and Band (delta, theta, alpha 1, alpha 2, alpha 3, beta 1, beta 2, and gamma). No statistically significant interaction among the factors Group, Hemisphere (Left, Right), ROI, and Band (p > 0.05).

**Figure SM6**. Mean values (± Standard Error Mean, SEM) of the intrahemispheric mean isolated lagged effective Coherence (iCoh)values computed in relation to a statistical ANOVA interaction (F = 1.91, p < 0.05) among the factors Group (Nold, ADD), Hemisphere (Left, Right), ROI (frontal, central, parietal, temporal and occipital) and Band (delta, theta, alpha 1, alpha 2, alpha 3, beta 1, beta 2, and gamma).

**Figure SM7**. Mean values (± Standard Error Mean, SEM) of the intrahemispheric absolute difference isolated lagged effective Coherence (iCoh)values computed in relation to a statistical ANOVA interaction (F = 2.13, p < 0.05) among the factors Group (Nold, ADD), Hemisphere (Left, Right), ROI (frontal, central, parietal, temporal and occipital) and Band (delta, theta, alpha 1, alpha 2, alpha 3, beta 1, beta 2, and gamma).

**Figure SM8**. Individual values (with the indication of mean across subjects ± Standard Error Mean, SEM, Log10 transformed) of the alpha2 and alpha3 interhemispheric Linear Lagged Connectivity showing statistically significant differences between ADD and Nold groups (see Figure 2). No statistically significant outliers according to Grubbs’ test were observed (p > 0.01).

**Figure SM9**. Individual values (with the indication of mean across subjects ± SEM, Log10 transformed) of the alpha2 and alpha3 intrahemispheric Linear Lagged Connectivity showing statistically significant differences between ADD and Nold groups (see Figure 3). No statistically significant outliers according to Grubbs’ test were observed (p > 0.01).

**Figure SM10**. Individual values (with the indication of mean across subjects ± SEM, Log10 transformed) of the global output and input isolated lagged effective coherence for degree and connector hubs in the alpha2 (left) and alpha3 (right) band. No statistically significant outliers according to Grubbs’ test (p < 0.0001) were observed.

**Figure SM11.** Mean values (± Standard Error Mean, SEM) of the normalized spectral power density values computed in relation to a statistical ANOVA interaction (F (28, 2100) = 6.97, p < 0.05) among the factors Group (Nold, ADD), ROI (frontal, central, parietal, temporal, and occipital) and Band (delta, theta, alpha 1, alpha 2, alpha 3, beta 1, beta 2, and gamma). Legend: the rectangles indicate statistically significant differences according to planned post-hoc test (Duncan’s; p < 0.05).

**Supplementary Materials Tables**

**Table SM1**

| ***Interhemispheric LLC*** | | ***Intrahemispheric LLC*** | |
| --- | --- | --- | --- |
| ***Statistical comparison (ADD ≠ Nold)*** | ***P-value*** | ***Statistical comparison (ADD ≠ Nold)*** | ***P-value*** |
| *Frontal delta* | 0.003987 | *Frontal alpha1* | 0.030777 |
| *Central alpha1* | 0.026886 | *Frontal alpha2* | 0.030548 |
| *Parietal delta* | 0.000137 | *Central alpha2* | 0.000002 |
| *Parietal alpha1* | 0.032089 | *Central alpha3* | 0.000025 |
| *Parietal alpha2* | 0.000246 | *Parietal alpha1* | 0.043862 |
| *Temporal alpha2* | 0.001209 | *Parietal alpha2* | 0.000002 |
| *Temporal alpha3* | 0.000028 | *Parietal alpha3* | 0.001242 |
| *Occipital delta* | 0.015961 | *Temporal alpha2* | 0.015931 |
| *Occipital theta* | 0.010405 | *Occipital alpha2* | 0.000072 |
|  |  | *Occipital alpha3* | 0.002349 |

**Table SM2**

| ***Degree hubs (scalp EEG electrodes) from LLC in alpha2 band*** | | | | | | |
| --- | --- | --- | --- | --- | --- | --- |
| ***Nodal Degree mean + 1SD***  ***(83^th^ percentile)*** | | | ***Nodal Degree***  ***80^th^ percentile*** | | | |
| **Threshold** | **Group** | **Degree Hubs** | **Threshold** | **Group** | **Degree Hubs** | |
| 0.1 | *ADD* | *P7, P8, O1* | 0.1 | *ADD* | *Fz, P7, P8, O1* | |
|  | *Nold* | *P3, P4, P7, P8, O2* |  | *Nold* | *P3, P7, P8, O2* | |
| 0.2 | *ADD* | *P7, P8, O1, O2* | 0.2 | *ADD* | *P7, P8, O1, O2* | |
|  | *Nold* | *P3, Pz, P7, P8, O2* |  | *Nold* | *Pz, P7, P8, O2* | |
| ***Nodal Degree***  ***70^th^ percentile*** | | | ***Nodal Degree mean + 1SEM***  ***(58^th^ percentile)*** | | | |
| **Threshold** | **Group** | **Degree Hubs** | **Threshold** | **Group** | **Degree Hubs** | |
| 0.1 | *ADD* | *Fz, P4, P7, P8, O1* | *0.1* | *ADD* | *Fz, P3, P4, Pz, P7, P8, O1, O2* | |
|  | *Nold* | *P3, P4, Pz, P7, P8, O2* |  | *Nold* | *Cz, P3, P4, Pz, P7, P8, O1, O2* | |
| 0.2 | *ADD* | *Fz, Pz, P7, P8, O1, O2* | *0.2* | *ADD* | *Fz, P4, Pz, P7, P8, O1, O2* | |
|  | *Nold* | *P3, Pz, P7, P8, O1, O2* |  | *Nold* | *P3, P4, Pz, P7, P8, O1, O2* |  |

**Table SM3**

| ***Degree hubs (scalp EEG electrodes) from LLC in alpha3 band*** | | | | | | |
| --- | --- | --- | --- | --- | --- | --- |
| ***Nodal Degree mean + 1SD***  ***(83^th^ percentile)*** | | | ***Nodal Degree***  ***80^th^ percentile*** | | | |
| **Threshold** | **Group** | **Degree Hubs** | **Threshold** | **Group** | **Degree Hubs** | |
| 0.1 | *ADD* | P7, P8, O1 | 0.1 | *ADD* | P7, P8, O1, O2 | |
|  | *Nold* | Pz, O1, O2 |  | *Nold* | P4, Pz, O1, O2 | |
| 0.2 | *ADD* | P7, P8, O1 | 0.2 | *ADD* | P7, P8, O1, O2 | |
|  | *Nold* | P4, Pz, O1, O2 |  | *Nold* | P4, Pz, O1, O2 | |
| ***Nodal Degree***  ***70^th^ percentile*** | | | ***Nodal Degree mean + 1SEM***  ***(58^th^ percentile)*** | | | |
| **Threshold** | **Group** | **Degree Hubs** | **Threshold** | **Group** | **Degree Hubs** | |
| 0.1 | *ADD* | Fz, Pz, P7, P8, O1, O2 | *0.1* | *ADD* | Fz, P4, Pz, P7, P8, O1, O2 | |
|  | *Nold* | Cz, P4, Pz, P8, O1, O2 |  | *Nold* | Cz, P3, P4, Pz, P7, P8, O1, O2 | |
| 0.2 | *ADD* | P3, Pz, P7, P8, O1, O2 | *0.2* | *ADD* | Fz, P3, Pz, P7, P8, O1, O2 | |
|  | *Nold* | P4, Pz, P7, P8, O1, O2 |  | *Nold* | Cz, P3, P4, Pz, P7, P8, O1, O2 |  |

**Table SM4**

| ***Degree hubs (scalp EEG electrodes) from mean iCoh in alpha2 band*** | | | | | | |
| --- | --- | --- | --- | --- | --- | --- |
| ***Nodal Degree mean + 1SD***  ***(83^th^ percentile)*** | | | ***Nodal Degree***  ***80^th^ percentile*** | | | |
| **Threshold** | **Group** | **Degree Hubs** | **Threshold** | **Group** | **Degree Hubs** | |
| 0.1 | *ADD* | P3, P4 | 0.1 | *ADD* | P3, P4, Cz, Pz | |
|  | *Nold* | P3, P4, Pz |  | *Nold* | P3, P4, Pz | |
| 0.2 | *ADD* | P3, P4 | 0.2 | *ADD* | P3, P4, Fz, Pz | |
|  | *Nold* | P3, P4, Pz |  | *Nold* | P3, P4, Fz, Pz | |
| ***Nodal Degree***  ***70^th^ percentile*** | | | ***Nodal Degree mean + 1SEM***  ***(58^th^ percentile)*** | | | |
| **Threshold** | **Group** | **Degree Hubs** | **Threshold** | **Group** | **Degree Hubs** | |
| 0.1 | *ADD* | C4, P3, P4, Fz, Cz, Pz | *0.1* | *ADD* | P3, P4, Cz, Pz | |
|  | *Nold* | P3, P4, Pz, O1, O2 |  | *Nold* | P3, P4, Pz, O1 | |
| 0.2 | *ADD* | P3, P4, Fz, Cz, Pz | *0.2* | *ADD* | P3, P4, Fz, Pz | |
|  | *Nold* | P3, P4, Fz, Pz, P8, O1 |  | *Nold* | P3, P4, Fz, Pz, P8, O1 |  |

**Table SM5**

| ***Degree hubs (scalp EEG electrodes) from mean iCoh in alpha3 band*** | | | | | | |
| --- | --- | --- | --- | --- | --- | --- |
| ***Nodal Degree mean + 1SD***  ***(83^th^ percentile)*** | | | ***Nodal Degree***  ***80^th^ percentile*** | | | |
| **Threshold** | **Group** | **Degree Hubs** | **Threshold** | **Group** | **Degree Hubs** | |
| 0.1 | *ADD* | P3, P4 | 0.1 | *ADD* | P3, P4, Cz, Pz | |
|  | *Nold* | P3, P4, Pz |  | *Nold* | P3, P4, Pz, O1 | |
| 0.2 | *ADD* | P3, P4 | 0.2 | *ADD* | C4, P3, P4, Pz | |
|  | *Nold* | P3, P4, Pz |  | *Nold* | P3, P4, Pz, O1 | |
| ***Nodal Degree***  ***70^th^ percentile*** | | | ***Nodal Degree mean + 1SEM***  ***(58^th^ percentile)*** | | | |
| **Threshold** | **Group** | **Degree Hubs** | **Threshold** | **Group** | **Degree Hubs** | |
| 0.1 | *ADD* | P3, P4, Cz, Pz, P7, O2 | *0.1* | *ADD* | P3, P4, Cz, Pz | |
|  | *Nold* | P3, P4, Fz, Pz, O1, O2 |  | *Nold* | P3, P4, Pz, O1 | |
| 0.2 | *ADD* | C4, P3, P4, Pz, P7, O2 | *0.2* | *ADD* | C4, P3, P4, Pz | |
|  | *Nold* | P3, P4, Fz, Pz, P8, O1 |  | *Nold* | P3, P4, Pz |  |

**Table SM6**

| ***Degree hubs (scalp EEG electrodes) from absolute difference iCoh in alpha2 band*** | | | | | | |
| --- | --- | --- | --- | --- | --- | --- |
| ***Nodal Degree mean + 1SD***  ***(83^th^ percentile)*** | | | ***Nodal Degree***  ***80^th^ percentile*** | | | |
| **Threshold** | **Group** | **Degree Hubs** | **Threshold** | **Group** | **Degree Hubs** | |
| 0.1 | *ADD* | P3, P4 | 0.1 | *ADD* | P3, P4, Cz, Pz | |
|  | *Nold* | P3, P4, Pz |  | *Nold* | P3, P4, Pz, O1 | |
| 0.2 | *ADD* | P3, P4 | 0.2 | *ADD* | P3, P4, Fz, Pz | |
|  | *Nold* | P3, P4, Pz |  | *Nold* | P3, P4, Pz, O1 | |
| ***Nodal Degree***  ***70^th^ percentile*** | | | ***Nodal Degree mean + 1SEM***  ***(58^th^ percentile)*** | | | |
| **Threshold** | **Group** | **Degree Hubs** | **Threshold** | **Group** | **Degree Hubs** | |
| 0.1 | *ADD* | P3, P4, Fz, Cz, Pz, P7 | *0.1* | *ADD* | P3, P4, Cz, Pz | |
|  | *Nold* | P3, P4, Pz, P8, O1 |  | *Nold* | P3, P4, Pz, O1 | |
| 0.2 | *ADD* | P3, P4, Fz, Cz, Pz | *0.2* | *ADD* | P3, P4, Fz, Pz | |
|  | *Nold* | P3, P4, Pz, P8, O1 |  | *Nold* | P3, P4, Fz, Pz, O1 |  |

**Table SM7**

| ***Degree hubs (scalp EEG electrodes) from absolute difference iCoh in alpha3 band*** | | | | | | |
| --- | --- | --- | --- | --- | --- | --- |
| ***Nodal Degree mean + 1SD***  ***(83^th^ percentile)*** | | | ***Nodal Degree***  ***80^th^ percentile*** | | | |
| **Threshold** | **Group** | **Degree Hubs** | **Threshold** | **Group** | **Degree Hubs** | |
| 0.1 | *ADD* | P3, P4 | 0.1 | *ADD* | C4, P3, P4, Pz | |
|  | *Nold* | P3, P4, Pz |  | *Nold* | P3, P4, Pz | |
| 0.2 | *ADD* | P3, P4 | 0.2 | *ADD* | C4, P3, P4, Pz | |
|  | *Nold* | P3, P4, Pz |  | *Nold* | P3, P4, Fz, Pz | |
| ***Nodal Degree***  ***70^th^ percentile*** | | | ***Nodal Degree mean + 1SEM***  ***(58^th^ percentile)*** | | | |
| **Threshold** | **Group** | **Degree Hubs** | **Threshold** | **Group** | **Degree Hubs** | |
| 0.1 | *ADD* | C4, P3, P4, Fz, Pz, P7 | *0.1* | *ADD* | P3, P4 | |
|  | *Nold* | P3, P4, Fz, Pz, P7, O1 |  | *Nold* | P3, P4, Pz | |
| 0.2 | *ADD* | C4, P3, P4, Pz, P7, O1 | *0.2* | *ADD* | C4, P3, P4, Pz | |
|  | *Nold* | P3, P4, Fz, Pz, P8, O1 |  | *Nold* | P3, P4, Fz, Pz |  |

**Table SM8**

| ***Connector and Provincial hubs (scalp EEG electrodes) from LLC in alpha2 band*** | | | | | | | |
| --- | --- | --- | --- | --- | --- | --- | --- |
| ***ND, PC and CC mean +/- 1SD***  ***(83^th^/16^th^ percentile)*** | | | | ***ND, PC and CC***  ***80^th^/20^th^ percentile*** | | | |
| **Threshold** | **Group** | **Connector Hub** | **Provincial Hub** | **Threshold** | **Group** | **Connector Hub** | **Provincial Hub** |
| 0.1 | *ADD* | - | - | 0.1 | *ADD* | - | - |
|  | *Nold* | - | - |  | *Nold* | - | - |
| 0.2 | *ADD* | - | - | 0.2 | *ADD* | O1 | - |
|  | *Nold* | P7 | - |  | *Nold* | P7 | - |
| ***ND, PC and CC***  ***70^th^/30^th^ percentile*** | | | | ***ND, PC and CC mean +/- 1SEM***  ***(58^th^/46^th^ percentile)*** | | | |
| **Threshold** | **Group** | **Connector Hub** | **Provincial Hub** | **Threshold** | **Group** | **Connector Hub** | **Provincial Hub** |
| 0.1 | *ADD* | O1 | *-* | 0.1 | *ADD* | P7, O1 | *-* |
|  | *Nold* | - | *-* |  | *Nold* | Cz, P8 | *Pz* |
| 0.2 | *ADD* | P8, O1 | *-* | 0.2 | *ADD* | P8, O1 | *Fp2* |
|  | *Nold* | P7 | *-* |  | *Nold* | P7, O1 | *-* |

**Table SM9**

| ***Connector and Provincial hubs (scalp EEG electrodes) from LLC in alpha3 band*** | | | | | | | |
| --- | --- | --- | --- | --- | --- | --- | --- |
| ***ND, PC and CC mean +/- 1SD***  ***(83^th^/16^th^ percentile)*** | | | | ***ND, PC and CC***  ***80^th^/20^th^ percentile*** | | | |
| **Threshold** | **Group** | **Connector Hub** | **Provincial Hub** | **Threshold** | **Group** | **Connector Hub** | **Provincial Hub** |
| 0.1 | *ADD* | P7 | - | 0.1 | *ADD* | P7 | - |
|  | *Nold* | - | - |  | *Nold* | - | - |
| 0.2 | *ADD* | - | - | 0.2 | *ADD* | - | - |
|  | *Nold* | - | *-* |  | *Nold* | - | *-* |
| ***ND, PC and CC***  ***70^th^/30^th^ percentile*** | | | | ***ND, PC and CC mean +/- 1SEM***  ***(58^th^/46^th^ percentile)*** | | | |
| **Threshold** | **Group** | **Connector Hub** | **Provincial Hub** | **Threshold** | **Group** | **Connector Hub** | **Provincial Hub** |
| 0.1 | *ADD* | Pz, P7, P8, O1 | - | 0.1 | *ADD* | Pz, P7, P8, O1, O2 | - |
|  | *Nold* | Cz | - |  | *Nold* | Cz | P8 |
| 0.2 | *ADD* | P7, P8, O1 | - | 0.2 | *ADD* | P7, P8, O1 | - |
|  | *Nold* | - | - |  | *Nold* | Cz | - |

**Table SM10**

| ***Connector and Provincial hubs (scalp EEG electrodes) from mean iCoh in alpha2 band*** | | | | | | | |
| --- | --- | --- | --- | --- | --- | --- | --- |
| ***ND, PC and CC mean +/- 1SD***  ***(83^th^/16^th^ percentile)*** | | | | ***ND, PC and CC***  ***80^th^/20^th^ percentile*** | | | |
| **Threshold** | **Group** | **Connector Hub** | **Provincial Hub** | **Threshold** | **Group** | **Connector Hub** | **Provincial Hub** |
| 0.1 | *ADD* | - | - | 0.1 | *ADD* | - | - |
|  | *Nold* | - | - |  | *Nold* | - | - |
| 0.2 | *ADD* | P3, P4 | - | 0.2 | *ADD* | P3, P4 | - |
|  | *Nold* | P3 | - |  | *Nold* | P3 | - |
| ***ND, PC and CC***  ***70^th^/30^th^ percentile*** | | | | ***ND, PC and CC mean +/- 1SEM***  ***(58^th^/46^th^ percentile)*** | | | |
| **Threshold** | **Group** | **Connector Hub** | **Provincial Hub** | **Threshold** | **Group** | **Connector Hub** | **Provincial Hub** |
| 0.1 | *ADD* | - | - | 0.1 | *ADD* | - | - |
|  | *Nold* | - | - |  | *Nold* | - | - |
| 0.2 | *ADD* | P3, P4 | - | 0.2 | *ADD* | P3, P4 | - |
|  | *Nold* | P3, P4 | - |  | *Nold* | P3, P4 | - |

**Table SM11**

| ***Connector and Provincial hubs (scalp EEG electrodes) from mean iCoh in alpha3 band*** | | | | | | | |
| --- | --- | --- | --- | --- | --- | --- | --- |
| ***ND, PC and CC mean +/- 1SD***  ***(83^th^/16^th^ percentile)*** | | | | ***ND, PC and CC***  ***80^th^/20^th^ percentile*** | | | |
| **Threshold** | **Group** | **Connector Hub** | **Provincial Hub** | **Threshold** | **Group** | **Connector Hub** | **Provincial Hub** |
| 0.1 | *ADD* | - | - | 0.1 | *ADD* | - | - |
|  | *Nold* | - | - |  | *Nold* | - | - |
| 0.2 | *ADD* | P4 | - | 0.2 | *ADD* | P3, P4 | - |
|  | *Nold* | P3, P4 | - |  | *Nold* | P3, P4 | - |
| ***ND, PC and CC***  ***70^th^/30^th^ percentile*** | | | | ***ND, PC and CC mean +/- 1SEM***  ***(58^th^/46^th^ percentile)*** | | | |
| **Threshold** | **Group** | **Connector Hub** | **Provincial Hub** | **Threshold** | **Group** | **Connector Hub** | **Provincial Hub** |
| 0.1 | *ADD* | - | - | 0.1 | *ADD* | P3 | - |
|  | *Nold* | - | - |  | *Nold* | - | - |
| 0.2 | *ADD* | P3, P4 | - | 0.2 | *ADD* | P3, P4 | - |
|  | *Nold* | P3, P4 | - |  | *Nold* | P3, P4 | - |

**Table SM12**

| ***Connector and Provincial hubs (scalp EEG electrodes) from absolute difference iCoh in alpha2 band*** | | | | | | | |
| --- | --- | --- | --- | --- | --- | --- | --- |
| ***ND, PC and CC mean +/- 1SD***  ***(83^th^/16^th^ percentile)*** | | | | ***ND, PC and CC***  ***80^th^/20^th^ percentile*** | | | |
| **Threshold** | **Group** | **Connector Hub** | **Provincial Hub** | **Threshold** | **Group** | **Connector Hub** | **Provincial Hub** |
| 0.1 | *ADD* | - | - | 0.1 | *ADD* | - | - |
|  | *Nold* | - | - |  | *Nold* | - | - |
| 0.2 | *ADD* | P4 | - | 0.2 | *ADD* | P4 | - |
|  | *Nold* | P3, P4 | - |  | *Nold* | P3, P4 | - |
| ***ND, PC and CC***  ***70^th^/30^th^ percentile*** | | | | ***ND, PC and CC mean +/- 1SEM***  ***(58^th^/46^th^ percentile)*** | | | |
| **Threshold** | **Group** | **Connector Hub** | **Provincial Hub** | **Threshold** | **Group** | **Connector Hub** | **Provincial Hub** |
| 0.1 | *ADD* | - | - | 0.1 | *ADD* | Cz | - |
|  | *Nold* | - | - |  | *Nold* | - | - |
| 0.2 | *ADD* | P4 | - | 0.2 | *ADD* | P3, P4 | - |
|  | *Nold* | P3, P4 | - |  | *Nold* | P3, P4, pz | - |

**Table SM13**

| ***Connector and Provincial hubs (scalp EEG electrodes) from absolute difference iCoh in alpha3 band*** | | | | | | | |
| --- | --- | --- | --- | --- | --- | --- | --- |
| ***ND, PC and CC mean +/- 1SD***  ***(83^th^/16^th^ percentile)*** | | | | ***ND, PC and CC***  ***80^th^/20^th^ percentile*** | | | |
| **Threshold** | **Group** | **Connector Hub** | **Provincial Hub** | **Threshold** | **Group** | **Connector Hub** | **Provincial Hub** |
| 0.1 | *ADD* | - | - | 0.1 | *ADD* | - | - |
|  | *Nold* | P4 | - |  | *Nold* | P4 | - |
| 0.2 | *ADD* | P4 | - | 0.2 | *ADD* | P4 | - |
|  | *Nold* | P3, P4 | - |  | *Nold* | P3, P4, Pz | - |
| ***ND, PC and CC***  ***70^th^/30^th^ percentile*** | | | | ***ND, PC and CC mean +/- 1SEM***  ***(58^th^/46^th^ percentile)*** | | | |
| **Threshold** | **Group** | **Connector Hub** | **Provincial Hub** | **Threshold** | **Group** | **Connector Hub** | **Provincial Hub** |
| 0.1 | *ADD* | - | - | 0.1 | *ADD* | - | - |
|  | *Nold* | P4 | - |  | *Nold* | P4 | - |
| 0.2 | *ADD* | P3, P4 | - | 0.2 | *ADD* | C4, P3, P4 | - |
|  | *Nold* | P3, P4, Pz | - |  | *Nold* | P3, P4, Pz | - |

**Table SM14**

| ***Connector and Provincial hubs (scalp EEG electrodes) from LLC in alpha2 band***  ***(Cole et al., 2015 approach)*** | | | | | | | |
| --- | --- | --- | --- | --- | --- | --- | --- |
| ***ND, PC and BC mean +/- 1SD***  ***(83^th^/16^th^ percentile)*** | | | | ***ND, PC and BC***  ***80^th^/20^th^ percentile*** | | | |
| **Threshold** | **Group** | **Connector Hub** | **Provincial Hub** | **Threshold** | **Group** | **Connector Hub** | **Provincial Hub** |
| 0.1 | *ADD* | *P8* | *-* | 0.1 | *ADD* | *P8* | *-* |
|  | *Nold* | *-* | *-* |  | *Nold* | *P7* | *-* |
| 0.2 | *ADD* | *-* | *-* | 0.2 | *ADD* | *O1, O2* | *-* |
|  | *Nold* | *P7, Pz* | *-* |  | *Nold* | *P7, Pz* | *-* |
| ***ND, PC and BC***  ***70^th^/30^th^ percentile*** | | | | ***ND, PC and BC mean +/- 1SEM***  ***(58^th^/46^th^ percentile)*** | | | |
| **Threshold** | **Group** | **Connector Hub** | **Provincial Hub** | **Threshold** | **Group** | **Connector Hub** | **Provincial Hub** |
| 0.1 | *ADD* | *Fz, P8, O1* | *-* | 0.1 | *ADD* | *Pz, P7, P8, O1* | *-* |
|  | *Nold* | *P3, Pz, P7* | *-* |  | *Nold* | *P3, Pz, P7, P8* | *-* |
| 0.2 | *ADD* | *Fz, P8, O1, O2* | *-* | 0.2 | *ADD* | *Fz, P8, O1, O2* | *-* |
|  | *Nold* | *Pz, P7, O1* | *-* |  | *Nold* | *P3, P4, Pz, P7, O1, O2* | *-* |

**Table SM15**

| ***Connector and Provincial hubs (scalp EEG electrodes) from LLC in alpha3 band***  ***(Cole et al., 2015 approach)*** | | | | | | | |
| --- | --- | --- | --- | --- | --- | --- | --- |
| ***ND, PC and BC mean +/- 1SD***  ***(83^th^/16^th^ percentile)*** | | | | ***ND, PC and BC***  ***80^th^/20^th^ percentile*** | | | |
| **Threshold** | **Group** | **Connector Hub** | **Provincial Hub** | **Threshold** | **Group** | **Connector Hub** | **Provincial Hub** |
| 0.1 | *ADD* | *P7, O1* | *-* | 0.1 | *ADD* | *P7, O1* | *-* |
|  | *Nold* | *O2* | *-* |  | *Nold* | *O1, O2* | *-* |
| 0.2 | *ADD* | *O1* | *-* | 0.2 | *ADD* | *P7, O1* | *-* |
|  | *Nold* | *P4, O1, O2* | *-* |  | *Nold* | *O1, O2* | *-* |
| ***ND, PC and BC***  ***70^th^/30^th^ percentile*** | | | | ***ND, PC and BC mean +/- 1SEM***  ***(58^th^/46^th^ percentile)*** | | | |
| **Threshold** | **Group** | **Connector Hub** | **Provincial Hub** | **Threshold** | **Group** | **Connector Hub** | **Provincial Hub** |
| 0.1 | *ADD* | *Fz, Pz, P7, P8, O1* | *-* | 0.1 | *ADD* | *Fz, P7, P8, O1* | *-* |
|  | *Nold* | *Cz, P4, Pz, O1, O2* | *-* |  | *Nold* | *Cz, P4, Pz, O1, O2* | *-* |
| 0.2 | *ADD* | *P7, P8, O1* | *-* | 0.2 | *ADD* | *Fz, P3, Pz, P7, P8, O1* | *-* |
|  | *Nold* | *P4, Pz, O1, O2* | *-* |  | *Nold* | *Cz, P4, Pz, P8, O1, O2* | *-* |

**Table SM16**

| ***Connector and Provincial hubs (scalp EEG electrodes) from mean iCoh in alpha2 band***  ***(Cole et al., 2015 approach)*** | | | | | | | |
| --- | --- | --- | --- | --- | --- | --- | --- |
| ***ND, PC and BC mean +/- 1SD***  ***(83^th^/16^th^ percentile)*** | | | | ***ND, PC and BC***  ***80^th^/20^th^ percentile*** | | | |
| **Threshold** | **Group** | **Connector Hub** | **Provincial Hub** | **Threshold** | **Group** | **Connector Hub** | **Provincial Hub** |
| 0.1 | *ADD* | P3, P4 | *-* | 0.1 | *ADD* | P3, P4, Cz | *-* |
|  | *Nold* | P3, P4, Pz | *-* |  | *Nold* | P3, P4, Pz | *-* |
| 0.2 | *ADD* | P3, P4 | *-* | 0.2 | *ADD* | P3, P4, Fz | *-* |
|  | *Nold* | P3, P4 | *-* |  | *Nold* | P3, P4 | *-* |
| ***ND, PC and BC***  ***70^th^/30^th^ percentile*** | | | | ***ND, PC and BC mean +/- 1SEM***  ***(58^th^/46^th^ percentile)*** | | | |
| **Threshold** | **Group** | **Connector Hub** | **Provincial Hub** | **Threshold** | **Group** | **Connector Hub** | **Provincial Hub** |
| 0.1 | *ADD* | P3, P4, Fz, Cz, Pz | *-* | 0.1 | *ADD* | P3, P4, Cz | *-* |
|  | *Nold* | P3, P4, Pz | *-* |  | *Nold* | P3, P4, Pz | *-* |
| 0.2 | *ADD* | P3, P4, Fz, Cz, Pz | *-* | 0.2 | *ADD* | P3, P4 | *-* |
|  | *Nold* | P3, P4, Cz, Pz | *-* |  | *Nold* | P3, P4,Fz, Pz | *-* |

**Table SM17**

| ***Connector and Provincial hubs (scalp EEG electrodes) from mean iCoh in alpha3 band***  ***(Cole et al., 2015 approach)*** | | | | | | | |
| --- | --- | --- | --- | --- | --- | --- | --- |
| ***ND, PC and BC mean +/- 1SD***  ***(83^th^/16^th^ percentile)*** | | | | ***ND, PC and BC***  ***80^th^/20^th^ percentile*** | | | |
| **Threshold** | **Group** | **Connector Hub** | **Provincial Hub** | **Threshold** | **Group** | **Connector Hub** | **Provincial Hub** |
| 0.1 | *ADD* | P3, P4 | *-* | 0.1 | *ADD* | P3, P4 | *-* |
|  | *Nold* | P3, P4, Pz | *-* |  | *Nold* | P3, P4, Pz | *-* |
| 0.2 | *ADD* | P3, P4 | *-* | 0.2 | *ADD* | P3, P4 | *-* |
|  | *Nold* | P3, P4, Pz | *-* |  | *Nold* | P3, P4, Pz | *-* |
| ***ND, PC and BC***  ***70^th^/30^th^ percentile*** | | | | ***ND, PC and BC mean +/- 1SEM***  ***(58^th^/46^th^ percentile)*** | | | |
| **Threshold** | **Group** | **Connector Hub** | **Provincial Hub** | **Threshold** | **Group** | **Connector Hub** | **Provincial Hub** |
| 0.1 | *ADD* | P3, P4, P7 | *-* | 0.1 | *ADD* | P3, P4 | *-* |
|  | *Nold* | P3, P4, Fz, Pz, O1 | *-* |  | *Nold* | P3, P4, Pz | *-* |
| 0.2 | *ADD* | P3, P4, Pz, O2 | *-* | 0.2 | *ADD* | P3, P4 | *-* |
|  | *Nold* | P3, P4, Fz, Pz, O1 | *-* |  | *Nold* | P3, P4, Pz | *-* |

**Table SM18**

| ***Connector and Provincial hubs (scalp EEG electrodes) from absolute difference iCoh in alpha2 band***  ***(Cole et al., 2015 approach)*** | | | | | | | |
| --- | --- | --- | --- | --- | --- | --- | --- |
| ***ND, PC and BC mean +/- 1SD***  ***(83^th^/16^th^ percentile)*** | | | | ***ND, PC and BC***  ***80^th^/20^th^ percentile*** | | | |
| **Threshold** | **Group** | **Connector Hub** | **Provincial Hub** | **Threshold** | **Group** | **Connector Hub** | **Provincial Hub** |
| 0.1 | *ADD* | P3, P4, Cz, Pz | *-* | 0.1 | *ADD* | P3, P4, Pz | *-* |
|  | *Nold* | P3, P4, Pz, P8, O1 | *-* |  | *Nold* | P3, P4, Pz | *-* |
| 0.2 | *ADD* | P3, P4 | *-* | 0.2 | *ADD* | P3, P4 | *-* |
|  | *Nold* | P3, P4, Pz, O1 | *-* |  | *Nold* | P3, P4, Pz | *-* |
| ***ND, PC and BC***  ***70^th^/30^th^ percentile*** | | | | ***ND, PC and BC mean +/- 1SEM***  ***(58^th^/46^th^ percentile)*** | | | |
| **Threshold** | **Group** | **Connector Hub** | **Provincial Hub** | **Threshold** | **Group** | **Connector Hub** | **Provincial Hub** |
| 0.1 | *ADD* | P3, P4, Cz, Pz | *-* | 0.1 | *ADD* | P3, P4, Cz, Pz | *-* |
|  | *Nold* | P3, P4, Pz | *-* |  | *Nold* | P3, P4, Pz | *-* |
| 0.2 | *ADD* | P3, P4, Cz, Pz | *-* | 0.2 | *ADD* | P3, P4 | *-* |
|  | *Nold* | P3, P4, Cz, Pz | *-* |  | *Nold* | P3, P4, Pz | *-* |

**Table SM19**

| ***Connector and Provincial hubs (scalp EEG electrodes) from absolute difference iCoh in alpha3 band***  ***(Cole et al., 2015 approach)*** | | | | | | | |
| --- | --- | --- | --- | --- | --- | --- | --- |
| ***ND, PC and BC mean +/- 1SD***  ***(83^th^/16^th^ percentile)*** | | | | ***ND, PC and BC***  ***80^th^/20^th^ percentile*** | | | |
| **Threshold** | **Group** | **Connector Hub** | **Provincial Hub** | **Threshold** | **Group** | **Connector Hub** | **Provincial Hub** |
| 0.1 | *ADD* | P3, P4 | *-* | 0.1 | *ADD* | P3, P4, Pz | *-* |
|  | *Nold* | P3, P4, Pz | *-* |  | *Nold* | P3, P4, Pz | *-* |
| 0.2 | *ADD* | P3, P4 | *-* | 0.2 | *ADD* | P3, P4, Cz, Pz | *-* |
|  | *Nold* | P3, P4, Pz | *-* |  | *Nold* | P3, P4, Pz | *-* |
| ***ND, PC and BC***  ***70^th^/30^th^ percentile*** | | | | ***ND, PC and BC mean +/- 1SEM***  ***(58^th^/46^th^ percentile)*** | | | |
| **Threshold** | **Group** | **Connector Hub** | **Provincial Hub** | **Threshold** | **Group** | **Connector Hub** | **Provincial Hub** |
| 0.1 | *ADD* | P3, P4, Pz, P7 | *-* | 0.1 | *ADD* | P3, P4, Pz | *-* |
|  | *Nold* | P3, P4, Pz, O1 | *-* |  | *Nold* | P3, P4, Pz | *-* |
| 0.2 | *ADD* | C4, P3, P4, Pz | *-* | 0.2 | *ADD* | P3, P4, Cz, Pz | *-* |
|  | *Nold* | P3, P4, Fz, Pz, O1 | *-* |  | *Nold* | P3, P4, Pz | *-* |

**Table SM20**

| ***Connector and Provincial hubs (scalp EEG electrodes) from LLC in alpha2 band***  ***(Power et al., 2013 approach)*** | | | | | | | |
| --- | --- | --- | --- | --- | --- | --- | --- |
| ***Within-module degree z-score and PC mean +/- 1SD***  ***(83^th^/16^th^ percentile)*** | | | | ***Within-module degree z-score and PC***  ***80^th^/20^th^ percentile*** | | | |
| **Threshold** | **Group** | **Connector Hub** | **Provincial Hub** | **Threshold** | **Group** | **Connector Hub** | **Provincial Hub** |
| 0.1 | *ADD* | P8 | - | 0.1 | *ADD* | P8 | - |
|  | *Nold* | P3 | - |  | *Nold* | P3, P7 | - |
| 0.2 | *ADD* | - | - | 0.2 | *ADD* | O1, O2 | - |
|  | *Nold* | P3, Pz, P7 | - |  | *Nold* | P7 | - |
| ***Within-module degree z-score and PC***  ***70^th^/30^th^ percentile*** | | | | ***Within-module degree z-score and PC mean +/- 1SEM***  ***(58^th^/46^th^ percentile)*** | | | |
| **Threshold** | **Group** | **Connector Hub** | **Provincial Hub** | **Threshold** | **Group** | **Connector Hub** | **Provincial Hub** |
| 0.1 | *ADD* | P8, O1 | - | 0.1 | *ADD* | P4, Pz, P7, P8, O1 | P3 |
|  | *Nold* | P3, Pz, P7 | - |  | *Nold* | P3, Cz, Pz, P7, P8 | - |
| 0.2 | *ADD* | P8, O1, O2 | - | 0.2 | *ADD* | P4, P8, O1, O2 | - |
|  | *Nold* | P3, Pz, P7, O1 | - |  | *Nold* | P3, P4, Pz, P7, O1, O2 | - |

**Table SM21**

| ***Connector and Provincial hubs (scalp EEG electrodes) from LLC in alpha3 band***  ***(Power et al., 2013 approach)*** | | | | | | | |
| --- | --- | --- | --- | --- | --- | --- | --- |
| ***Within-module degree z-score and PC mean +/- 1SD***  ***(83^th^/16^th^ percentile)*** | | | | ***Within-module degree z-score and PC***  ***80^th^/20^th^ percentile*** | | | |
| **Threshold** | **Group** | **Connector Hub** | **Provincial Hub** | **Threshold** | **Group** | **Connector Hub** | **Provincial Hub** |
| 0.1 | *ADD* | P7, O1 | - | 0.1 | *ADD* | P7, O1 | - |
|  | *Nold* | P4, O1, O2 | - |  | *Nold* | P4, O1, O2 | - |
| 0.2 | *ADD* | O1 | - | 0.2 | *ADD* | O1 | - |
|  | *Nold* | O1, O2 | - |  | *Nold* | O1, O2 | - |
| ***Within-module degree z-score and PC***  ***70^th^/30^th^ percentile*** | | | | ***Within-module degree z-score and PC mean +/- 1SEM***  ***(58^th^/46^th^ percentile)*** | | | |
| **Threshold** | **Group** | **Connector Hub** | **Provincial Hub** | **Threshold** | **Group** | **Connector Hub** | **Provincial Hub** |
| 0.1 | *ADD* | P4, Pz, P7, P8, O1 | - | 0.1 | *ADD* | P4, Pz, P7, P8, O1, O2 | - |
|  | *Nold* | P4, Pz, O1, O2 | P8 |  | *Nold* | P3, P4, Pz, O1, O2 | P8 |
| 0.2 | *ADD* | Pz, P7, P8, O1 | - | 0.2 | *ADD* | P3, Pz, P7, P8, O1 | - |
|  | *Nold* | P4, Pz, O1, O2 | - |  | *Nold* | P4, Pz, P8, O1, O2 | - |

**Table SM22**

| ***Connector and Provincial hubs (scalp EEG electrodes) from mean iCoh in alpha2 band***  ***(Power et al., 2013 approach)*** | | | | | | | |
| --- | --- | --- | --- | --- | --- | --- | --- |
| ***Within-module degree z-score and PC mean +/- 1SD***  ***(83^th^/16^th^ percentile)*** | | | | ***Within-module degree z-score and PC***  ***80^th^/20^th^ percentile*** | | | |
| **Threshold** | **Group** | **Connector Hub** | **Provincial Hub** | **Threshold** | **Group** | **Connector Hub** | **Provincial Hub** |
| 0.1 | *ADD* | P3, P4 | - | 0.1 | *ADD* | P3, P4 | - |
|  | *Nold* | P3, P4, Pz | - |  | *Nold* | P3, P4, Pz, O1 | - |
| 0.2 | *ADD* | P3, P4 | - | 0.2 | *ADD* | P3, P4 | - |
|  | *Nold* | P3, P4, Pz | - |  | *Nold* | P3, P4, Pz | - |
| ***Within-module degree z-score and PC***  ***70^th^/30^th^ percentile*** | | | | ***Within-module degree z-score and PC mean +/- 1SEM***  ***(58^th^/46^th^ percentile)*** | | | |
| **Threshold** | **Group** | **Connector Hub** | **Provincial Hub** | **Threshold** | **Group** | **Connector Hub** | **Provincial Hub** |
| 0.1 | *ADD* | P3, P4, Fz, Cz, Pz | *-* | 0.1 | *ADD* | P3, P4, Cz, Pz | *-* |
|  | *Nold* | P3, P4, Pz, P8, O1 | *-* |  | *Nold* | P3, P4, Pz, O1 | *-* |
| 0.2 | *ADD* | P3, P4, Cz, Pz | *-* | 0.2 | *ADD* | P3, P4, O1 | *-* |
|  | *Nold* | P3, P4, Fz, Pz, P8 | *-* |  | *Nold* | P3, P4, Fz, Pz, P8 | *-* |

**Table SM23**

| ***Connector and Provincial hubs (scalp EEG electrodes) from mean iCoh in alpha3 band***  ***(Power et al., 2013 approach)*** | | | | | | | |
| --- | --- | --- | --- | --- | --- | --- | --- |
| ***Within-module degree z-score and PC mean +/- 1SD***  ***(83^th^/16^th^ percentile)*** | | | | ***Within-module degree z-score and PC***  ***80^th^/20^th^ percentile*** | | | |
| **Threshold** | **Group** | **Connector Hub** | **Provincial Hub** | **Threshold** | **Group** | **Connector Hub** | **Provincial Hub** |
| 0.1 | *ADD* | P3, P4 | - | 0.1 | *ADD* | P3, P4, Pz | - |
|  | *Nold* | P3, P4 | - |  | *Nold* | P3, P4, Pz, O1 | - |
| 0.2 | *ADD* | P3, P4 | - | 0.2 | *ADD* | P3, P4, Pz | - |
|  | *Nold* | P3, P4, Pz | - |  | *Nold* | P3, P4, Pz | - |
| ***Within-module degree z-score and PC***  ***70^th^/30^th^ percentile*** | | | | ***Within-module degree z-score and PC mean +/- 1SEM***  ***(58^th^/46^th^ percentile)*** | | | |
| **Threshold** | **Group** | **Connector Hub** | **Provincial Hub** | **Threshold** | **Group** | **Connector Hub** | **Provincial Hub** |
| 0.1 | *ADD* | P3, P4, Cz, Pz | *-* | 0.1 | *ADD* | P3, P4, Cz, Pz | *-* |
|  | *Nold* | P3, P4, Pz, O1 | *-* |  | *Nold* | P3, P4, Pz, O1 | *-* |
| 0.2 | *ADD* | P3, P4, Pz, P7 | *-* | 0.2 | *ADD* | P3, P4, Fz, Pz, P7 | *-* |
|  | *Nold* | P3, P4, Fz, Pz, O1 | *-* |  | *Nold* | P3, P4, Fz, Pz | *-* |

**Table SM24**

| ***Connector and Provincial hubs (scalp EEG electrodes) from absolute difference iCoh in alpha2 band***  ***(Power et al., 2013 approach)*** | | | | | | | |
| --- | --- | --- | --- | --- | --- | --- | --- |
| ***Within-module degree z-score and PC mean +/- 1SD***  ***(83^th^/16^th^ percentile)*** | | | | ***Within-module degree z-score and PC***  ***80^th^/20^th^ percentile*** | | | |
| **Threshold** | **Group** | **Connector Hub** | **Provincial Hub** | **Threshold** | **Group** | **Connector Hub** | **Provincial Hub** |
| 0.1 | *ADD* | P3, P4 | - | 0.1 | *ADD* | P3, P4 | - |
|  | *Nold* | P3, P4, Pz | - |  | *Nold* | P3, P4, Pz | - |
| 0.2 | *ADD* | P3, P4 | - | 0.2 | *ADD* | P3, P4 | - |
|  | *Nold* | P3, P4 | - |  | *Nold* | P3, P4, Pz | - |
| ***Within-module degree z-score and PC***  ***70^th^/30^th^ percentile*** | | | | ***Within-module degree z-score and PC mean +/- 1SEM***  ***(58^th^/46^th^ percentile)*** | | | |
| **Threshold** | **Group** | **Connector Hub** | **Provincial Hub** | **Threshold** | **Group** | **Connector Hub** | **Provincial Hub** |
| 0.1 | *ADD* | P3, P4, Pz | *-* | 0.1 | *ADD* | P3, P4, Pz | *-* |
|  | *Nold* | P3, P4, Pz, P7, O1 | *-* |  | *Nold* | P3, P4, Pz, O1 | *-* |
| 0.2 | *ADD* | P3, P4, Cz, Pz | *-* | 0.2 | *ADD* | P3, P4, Pz, P7 | *-* |
|  | *Nold* | P3, P4, Pz, P8, O1 | *-* |  | *Nold* | P3, P4, Pz | *-* |

**Table SM25**

| ***Connector and Provincial hubs (scalp EEG electrodes) from absolute difference iCoh in alpha3 band***  ***(Power et al., 2013 approach)*** | | | | | | | |
| --- | --- | --- | --- | --- | --- | --- | --- |
| ***Within-module degree z-score and PC mean +/- 1SD***  ***(83^th^/16^th^ percentile)*** | | | | ***Within-module degree z-score and PC***  ***80^th^/20^th^ percentile*** | | | |
| **Threshold** | **Group** | **Connector Hub** | **Provincial Hub** | **Threshold** | **Group** | **Connector Hub** | **Provincial Hub** |
| 0.1 | *ADD* | P3, P4 | - | 0.1 | *ADD* | P3, P4, Pz | - |
|  | *Nold* | P3, P4, Pz | - |  | *Nold* | P3, P4, Pz, O1 | - |
| 0.2 | *ADD* | P3, P4 | - | 0.2 | *ADD* | P3, P4, Pz | - |
|  | *Nold* | P3, P4, Pz | - |  | *Nold* | P3, P4, Pz | - |
| ***Within-module degree z-score and PC***  ***70^th^/30^th^ percentile*** | | | | ***Within-module degree z-score and PC mean +/- 1SEM***  ***(58^th^/46^th^ percentile)*** | | | |
| **Threshold** | **Group** | **Connector Hub** | **Provincial Hub** | **Threshold** | **Group** | **Connector Hub** | **Provincial Hub** |
| 0.1 | *ADD* | P3, P4, Pz, P8 | *-* | 0.1 | *ADD* | P3, P4, Pz | *-* |
|  | *Nold* | P3, P4, Pz, O1 | *-* |  | *Nold* | P3, P4, Pz, O1 | *-* |
| 0.2 | *ADD* | P3, P4, Pz | *-* | 0.2 | *ADD* | P3, P4, Pz | *-* |
|  | *Nold* | P3, P4, Fz, Pz, O1 | *-* |  | *Nold* | P3, P4, Fz, Pz, O1 | *-* |
